# Time-related multivariate strategy for the comprehensive evaluation of microbial chemical data

**DOI:** 10.1007/s11306-022-01896-6

**Published:** 2022-05-24

**Authors:** Denise M. Selegato, Thamires R. Freitas, Marcos Pivatto, Amanda D. Pivatto, Alan C. Pilon, Ian Castro-Gamboa

**Affiliations:** 1 Nucleus of Bioassays, Biosynthesis, and Ecophysiology of Natural Products (NuBBE), Institute of Chemistry, São Paulo State University, Araraquara, SP Brazil; 2 Núcleo de Pesquisa em Compostos Bioativos (NPCBio), Instituto de Química, Universidade Federal de Uberlândia, Uberlândia, MG Brazil; 3 Núcleo de Pesquisa em Produtos Naturais e Sintéticos (NPPNS), Faculdade de Ciências Farmacêuticas de Ribeirão Preto, Universidade de São Paulo, Ribeirão Preto, SP Brazil; 4 Present Address: Zimmermann Group, Structural and Computational Biology Unit, European Molecular Biology Laboratory (EMBL), Heidelberg, Germany

**Keywords:** Biotransformation, Microbial-specific multivariate data analyses, Defense response, Toxic piperidine alkaloids, *Fusarium oxysporum*, PLS discriminant analysis, PLS regression

## Abstract

**Introduction:**

In microbial metabolomics, the use of multivariate data analysis (MDVA) has not been comprehensively explored regarding the different techniques available and the information that each gives about the metabolome. To overcome these limitations, here we show the use of *Fusarium oxysporum* cultured in the presence of exogenous alkaloids as a model system to demonstrate a comprehensive strategy for metabolic profiling.

**Matherials and methods:**

*F. oxysporum* was harvested on different days of incubation after alkaloidal addition, and the chemical profiles were compared using LC–MS data and MDVA. We show significant innovation to evaluate the chemical production of microbes during their life cycle by utilizing the full capabilities of Partial Least Square (PLS) with microbial-specific modeling that considers incubation days, media culture availability, and growth rate in solid media.

**Results and Discusscion:**

Results showed that the treatment of the Y-data and the use of both PLS regression and discrimination (PLSr and PLS-DA) inferred complemental chemical information. PLSr revealed the metabolites that are produced/consumed during fungal growth, whereas PLS-DA focused on metabolites that are only consumed/produced at a specific period. Both regression and classificatory analysis were equally important to identify compounds that are regulated and/or selectively produced as a response to the presence of the alkaloids. Lastly, we report the annotation of analogs from the piperidine alkaloids biotransformed by *F. oxysporum* as a defense response to the toxic plant metabolites. These molecules do not show the antimicrobial potential of their precursors in the fungal extracts and were rapidly produced and consumed within 4 days of microbial growth.

**Supplementary Information:**

The online version contains supplementary material available at 10.1007/s11306-022-01896-6.

## Introduction

In microbes, secondary metabolites are generated by the expression of biosynthetic gene clusters (BGCs), a set of genes that produce complex molecules from simple protein precursors. Nevertheless, most of this undiscovered reservoir of biosynthetic pathways are not active under typical laboratory growth conditions, meaning that a much broader range of metabolites could be produced if these silent genes are induced by whatever methods. In the last decade, approaches that focus on metabolite elicitation in microbes have made considerable advances using pattern recognition and metabolomics to monitor the activation of cryptic genes. All these methodologies aim to increase the induction of silenced clusters using different mechanisms that range from co-cultivation to the use of elicitor molecules (Hibbing et al., 2010; Moon et al. 2019b; Seyedsayamdost, 2014). Contrarily to gene-based methodologies, the evaluation of gene modulation is performed at the metabolomic level and has provided important information on the ecological and biological properties of microbial systems as well as the mechanism of chemical regulation at the biosynthetic level. In principle, all these methodologies are readily scalable to evaluate a large number of strains and have shown massive success in finding promising products of silent BGCs (Craney et al., 2012).

In microbial metabolomics, the broad dynamic range and diversity of metabolites still hamper the identification and biological correlation of bioactive compounds. As a result, the selection of the analytical platform needs to consider not only reproducibility but also metabolite coverage and sensitivity. Most metabolomics studies reported in the literature have been performed using mass spectrometry (MS) coupled with separation techniques, such as liquid and gas chromatography. The use of these combined techniques minimizes their technical limitations and increases separation, detection, stability, sensitivity, and amplitude in the detection range. Moreover, MS data contains definitive characteristics of a molecule deduced from accurate mass, fragmentation patterns, and isotope distribution, enabling chemical annotation of unknown analogs and other novel metabolites not available in current databases (Huber et al., 2021; Wang et al., 2016).

Despite the analytical advantages of MS-based metabolomics, in practice, it is difficult to identify a given compound reliably when solely using precursors because of the inflated number of results returned in databases. Often, to find meaningful information, specialized forms of data analysis are required. One approach that has been extensively applied in the last decade involves multivariate data analysis (MVDA), in which spectral features contributing the most to sample variation or separation are statistically identified and used for further chemical evaluation (Worley & Powers, 2013). MDVA has been used with tremendous success in metabolomics datasets applied to species profiling, quality control, genetic transformations, drug development, chemical ecology, and the understanding of the biochemical function and taxonomic classification (Selegato et al., 2019).

Chemometric techniques have been unadvisedly used in microbial metabolomics. However, there is still a lack of information related to the different types of techniques available and the knowledge that each gives. Particularly, little is done regarding the role that culture media availability and growth rate play in the modeling of the supervised methods. Hence, the present work aims to use the response of *Fusarium oxysporum* to the toxic piperidine alkaloids as a model to show a simple strategy for the comprehensive profiling of these targeted compounds, biotransformed analogues, and/or induced/suppressed molecules.

To show such methodology, *F. oxysporum* was cultivated in solid media and harvested on days 1, 4, 8, 12, and 16 after alkaloidal addition. The chemical profile was compared with control samples (only *Fusarium without alkaloids on the culture media*) using LC–MS data and multivariate data analyses (MDVA). For the comparative MDVA, we show significant innovation to evaluate the chemical production of microbes during their life cycle by utilizing the full capabilities offered by Partial Least Square (both PLS-DA and PLSr) with microbial-specific modeling that considers incubation days, media culture availability, and growth rate. In addition to detailing the parameters used for unsupervised and supervised methods, we highlight the advantages and disadvantages of each chemometric technique in microbial metabolomics, hoping to increase the readers' ability to determine microbial biomarkers and distinguish them with statistically validated confidence.

The selection of the piperidine alkaloids (–)-cassine and (–)-spectaline was mainly because *Fusarium oxysporum* LC055797.1 was isolated from the rhizosphere community of *Senna spectabilis* (Fabaceae). This plant is known to produce these toxic metabolites as major and most abundant compounds in their leaves, stems, and roots (Selegato et al., 2017). To date, it remains unknown the ecological outcome of the interaction of these abundant compounds and the microbiome that inhabits the plant, as well as the mechanism this strain uses to tolerate toxic metabolites. Furthermore, there is a notion that exogenous toxic molecules at subinhibitory concentrations could act as modulators of silent gene clusters in fungi and bacteria and, therefore, could be used for the discovery of new secondary metabolites in these microbial systems (Moon et al. 2019b; Xu et al., 2017). For instance, many studies have shown the potential of well-known clinical antibiotics to increase the production of new (cryptic) antibiotic molecules by the modulation of biosynthetic gene clusters (BGCs) (Moon et al., 2019a, 2019b; Zhang & Seyedsayamdost, 2020). In this sense, the use of toxic alkaloids could shed light on the possible metabolomic modulation of the fungus biosynthetic pathways. Lastly, although these piperidine alkaloids show high toxicity, they have also been linked to several biological properties, encouraging the exploration of *F. oxysporum* biosynthetic machinery for the production of potential analogues that causes less damage and are difficult to obtain synthetically (Pereira et al., 2016; Selegato et al., 2017; Viegas Júnior et al., 2004, 2007).

## Materials and methods

### Incubation parameters and metabolite extraction in solid media

Plugs of the selected fungi were grown in Petri dishes containing 20.0 mL of Czapek-Agar (Sigma Aldrich, Brazil) and the selected concentration of the piperidine alkaloid mixture (50 µg.mL^–1^). A negative control sample was prepared by the inoculation of the fungus in 20.0 mL of Czapek-Agar (NaNO_3_, 1.5 g.L^−1^; KH_2_PO_4_, 0.5 g.L^−1^; MgSO_4_, 0.25 g.L^−1^; FeSO_4_. 7H_2_O, 0.025 g.L^–1^; KCl, 2.5 g.L^–1^; D-glucose, 30.0 g.L^–1^ and agarose 20.0 g.L^–1^) without the alkaloids. These controls were incubated under the same conditions as all feeding experiments.

A 10 mm agar plug of a fungal pre-culture was inoculated in the center of a 9 cm petri dish for every experiment, and all plates were incubated at 25 °C for, respectively, 1, 4, 8, 12, and 16 days, with 12-h light cycles. For the metabolite extraction, the plates were excised with a razor blade in 3 × 1 cm pieces, transferred to a mortar, frozen with liquid nitrogen, and ground. The grained material was then resuspended in 200 mL of distilled water, sonicated for 45 min, vacuum filtered to remove agar and mycelia, and extracted with three portions of ethyl acetate (3 × 100 mL). The resulting organic solutions were then dehydrated with 20 g of anhydrous sodium sulfate (≥ 99%, NaSO_4_, Sigma Aldrich), vacuum filtrated, and evaporated to dryness under nitrogen.

### High-performance HR-LC-DAD-ESI–MS—data acquisition

Separation of compounds was performed in a Shimadzu Class-LC 10, CBM 20A controller. For the LC separation, a C18 Kinetex column (100 × 2.1 mm, 2.6 µm, 100 Å) was used at 35 °C, flow rate at 0.40 mL.min^–1^, and a UV-DAD detector operating between 190 to 600 nm. The chromatographic method was based on the de Vos and Lisec studies (De Vos et al., 2007; Lisec et al., 2006; Selegato et al., 2019). The injection volume was 5 mL. The mobile phase was composed of H_2_O (A) and methanol (B), both with 0.1% (v/v) formic acid, followed the gradient of: 5–35% B (23 min); 35–100% B (17 min), holding in 100% B for 8 min followed reducing from 100–5% B (2 min) and maintained initial condition (5% B) for 10 min, totaling 60 min of analysis.

The MS experiments were carried out in a micrOTOF-QII mass spectrometer (Bruker Daltonics, Bruker) using electrospray ionization (ESI) as ionization source and Time of Flight (TOF) analyzer in the positive ionization mode. The acquisition parameters were capillary temperature at 200 °C, N_2_ as a drying gas at 4.5 bar, spectral acquisition rate of 1.5 spectra per second, the flow of 9.0 L.min^–1^, and capillary and cone voltage of 4500 V and 500 V, respectively. The spectra were acquired between 100–1500 Da.

For high-resolution mass analysis, an internal standard of sodium trifluoroacetate (NaTFA solution at four mg.mL^–1^) was injected at the beginning and the end of each run (*m/z* 158.9640 for [M + Na] +). The exact mass calibration was automatically performed for every 10 injections. Organic solvent samples were also analyzed every 10 injections. The analysis of OS samples provided identification of impurities on the organic solvents or extraction procedure and permitted the removal of carryover contamination. System performance and stability were evaluated by the injection of QC samples consisted of a pool of equal volume of all samples used in the study.

### Data treatment and multivariate data analyses (MDVA)

For the statistical experiments, we have used the Total Ion Chromatogram (TIC) of the fungi with alkaloidal feeding (coded as B) and the control without feeding (coded as F) at different incubation periods. LC-data was converted from the proprietary Bruker file formats to the universal format of .mzXML using MassConvert (ProteoWizard) and these data was uploaded in MZMine2 for further preprocessing (Pluskal et al., 2010). This software performs peak detection, isotopic grouping, deconvolution, alignment and data filtering, providing a data matrix where the measured intensity, mass-to-charge ratio (*m/z*) and retention time (RT) are defined for each sample. Retention time (RT) windows and mass tolerances were determined for each analyzed set based on the data of selected chromatographic peaks. To minimize mass redundancy and enhance the true molecular feature selection, only intensities that showed significant statistical similarity within the same incubation day were chosen for the study. These regions include all the most abundant signals from the TIC, as well as other low-intensity signals. Lastly, features with relative standard deviation higher than 25% were discarded because of their unacceptable variability in the QC samples. The final matrix represented the intensity of 25 bins (reduced from 745 data points) over the 36 sample entries (three replicates for each sample plus the mean value for each class). The selected bins, the ANOVA test, and the entire chromatograms can be seen in Figure SM3 from the Supplementary Materials.

The final matrix was automatically reduced to ASCII files and imported to MATLAB software (Mathworks, Natick, MA). Further data preprocessing included the removal of the edges (only data with *t*_R_ between 11.5 and 31 min were used), baseline correction by Automatic Whittaker Filter (asymmetry of 0.001 and lambda 100), total area normalization, G-log transformation (lambda of 10^–1^) and autoscaling. Autoscaling was done to ensure that all peaks have the same weight (i.e., same importance in the analysis). Preprocessing was optimized based on trial-and-error of different parameters and functions. A visual comparison between the raw data and the final processed matrix is available as Figure SM4 from the Supplementary Material.

After preprocessing, the ionizable media compounds from the Czapek culture media were identified and accounted for by annotating a reference peak list during each experimental run (referred hereafter as CM). To account for instrument noise, we excluded signals that had a signal-to-noise ratio below five and retained only the ones occurring in a minimum of 75% of replicates. Contaminants were removed based on the organic solvent (OS) filtration and several features were excluded for unacceptable variability. Remaining variables were evaluated by multivariate statistical analysis.

MDVA was performed by unsupervised and supervised methods, and all analyses were calculated using the PLS Toolbox under the MATLAB environment (Eigenvector Research Inc., Wenatchee, WA). Hierarchical Clustering Analysis (HCA) was performed to observe the similarity (near) or dissimilarity (distant) between the samples and was calculated using ward distance for linkage and Euclidean distance as the pairwise distance. Euclidian distance gives the absolute distance between clusters and is currently the most common similarity measurement for this type of analysis. Similarly, Principal Component Analysis (PCA) was used as an explorative unbiased technique to evaluate the variables’ tendency. PCA was performed using the singular value decomposition (SVD) algorithm applied at a 95% confidence interval.

The supervised methods included both Partial Least Square regression (PLSr) and PLS Discriminant Analysis (PLS-DA). PLSr was validated for each data type (*Fusarium* with and without feeding, respectively). However, it was not validated using the entire matrix. In contrast, PLS-DA was only validated for the feeding samples using a four-class model. For both analyses, cross-validation (CV) was used to determine the number of principal components (PCs) needed to build the model and test its prediction capacity. This was done by leave-one-out methodology (also known as an n-fold CV), which is applied once for each sample and consists of using all samples but one as a training set, followed by a single-item test set using the excluded sample.

The number of PCs chosen for the model was based on the comparison between the PCs distribution and considers the lowest average root mean squared error of calibration (RMSEC) trends and the average root mean squared error of cross-validation (RMSECV) values. The ability to test whether the model could predict the class labels (also known as prediction accuracy) was done by the evaluation of the sum of squares captured by the model (R2) and the cross-validation (R2-CV or Q2). A permutation test was also applied for both supervised methods with 1000 interactions to identify the overfitting of the model. This is done by checking whether your model is significantly different from the null models created by randomly shuffling the class label (Y-vector). An evaluation was conducted by the empirical p-values, as well as the plot of Sum Squared Y (SSQY) versus Y-block correlation and a table of “Probability of Model Insignificance”. P‐values below 0.05 were considered statistically significant.

Variable Importance in Projection (VIP) was used to estimate the importance of each variable in both PLSr and PLS-DA. This scoring system represents the weighted sum of the squared correlations between the components and the original variables, in which the weight corresponds to the percentage of explained variance. The *vip* function of the PLS Toolbox was used to identify the Variable Importance in Projection (VIP) scores for each class. Variables with VIP scores higher than one can be considered important to the model, where, on the other hand, the variables with VIP scores lower than one has a low influence. Lastly, the performance of the models was assessed by the receiver operating characteristic (ROC) curve and the area under the curve (AUC). For this, specificity (true negative rate), sensitivity (true positive rate), and the optimal threshold value were compared along with the confusion matrices for each group to visualize the performance of the model.

### Identification of differential metabolites

Bruker Compass DataAnalysis 4.1 was used to establish the molecular formula from the experimental high-resolution mass and isotope pattern. The identification of molecular components was achieved through comparative searches on available databases for MS data, such as MassBank, Metlin, and MoNA. Additional MS/MS analysis was only acquired when necessary. We have also used other experimental information, such as retention time, UV absorption and height proportions between the [M + H] + and the [M + Na] + ions in order to assign tentative identification. In the cases in which annotation was not possible, scientific literature was consulted to infer the molecular classes and other important chemical features of the molecule. Finally, the mass error of all the candidates was equal or lower than 5 ppm.

### Bioassays

#### Antimicrobial assay

Antimicrobial activity was examined against two Gram-positive strains (*Bacillus cereus* and *Staphylococcus aureus*) and two gram-negative strains (*Pseudomonas fluorescens* and *Escherichia coli)* purchased from The Netherlands Culture Collection of Bacteria. The antimicrobial assay was performed according to the Clinical and Laboratory Standards Institute (CLSI) guidelines. Spectinomycin was used as a positive control at a concentration of 100 mg.mL^–1^. Bacteria were incubated at 37 °C in growth medium Mueller–Hinton Broth medium (MHB) (Sigma-Aldrich) until an optical density (OD) of 0.1. The cells were diluted to 10^6^ CFU.mL^−1^ before inoculation in a 96-well plate. Stock solutions of alkaloids and isolated compounds were prepared in 100% dimethyl sulfoxide (DMSO) at the concentration of 25 mg.mL^–1^ and stored at –20 °C until use. Each stock solution was diluted in serial dilutions of factor 2 with broth medium, reaching concentrations in the range of 512 to 64 µg.mL^−1^ before use. A volume of 100 µL of the broth containing the test bacteria was aliquoted to each well of the microtiter plate. The minimum inhibitory concentration (MIC) was determined after incubation at 30 °C overnight. The MIC was recorded as the highest dilution concentration of the plant extract resulting in growth inhibition (absence of turbidity) of the bacteria.

#### Antifungal evaluation by disc diffusion assay (DDA)

To address the toxicity of the exogenous compounds in *F. oxysporum,* the fungi was submitted to a disc diffusion assay using different alkaloidal concentrations, determining the highest concentration that could be used without being harmful to the microbe (i.e., subinhibitory concentration). For that, the spore solution of *F. oxysporum* was obtained by growing a 10 mm agar plug of the selected fungi in Petri dishes containing 20.0 ml of Oatmeal-Agar (Difco, Detroit) (Oatmeal 60 g.L^–1^; Bacto Agar 12.5 g.L^–1^). The fungal pre-culture was inoculated in the center of a 9 cm petri dish, and all plates were incubated at 25 °C for nine days in the dark. After incubation, 10.0 ml of sterile water was added to the fungal cultures, scraped with a plastic smear loop, and transferred to another petri dish. This procedure has been repeated a total of 5 times, and the final solution was vortexed for one minute and left to rest for 15 min to allow the particles to settle down. An automatic cell counter was used to determine the number of cells in the resulting spore solution, following the manufacturer’s instructions. Then, 10 µL of the upper supernatant of the spore solution was loaded onto the TC20 system (Bio-Rad, Hercules) counting slides, and the number of cells was determined with a TC20 automated cell counter (Bio-Rad, Hercules). All solutions were diluted with sterile water until the final concentration of 1 × 10^6^ cells.mL^–1^, and the remaining were stored at –80 °C.

Preparation of the solid media for the disc diffusion assay was conducted by the addition of 20.0 mL of Czapek-Agar (Sigma Aldrich) and 500 µL of the *F. oxysporum* spore solution at a cell concentration of 1 × 10^6^ cells.mL^–1^. After solidification of the solid media with the spores, four wells of 7 mm were pierced in each plate, with the aid of a modified Pasteur pipette, and the alkaloidal solution was added in triplicates with concentrations of 500, 300 200, 150, 100, and 50 µg.mL^–l^. All the alkaloidal solutions were prepared in ethyl acetate. The fourth well of each plate was used as a negative blank, containing only ethyl acetate. Antifungal susceptibility of *F. oxysporum* against six different concentrations of the alkaloid was determined based on the measurement of the inhibition zone diameters (in cm) after ten days of incubation. Pictures of the plates are available in Figure SM2 in the Supplementary Material.

## Results and discussion

### Chemical fingerprinting of *F. oxysporum*—unsupervised multivariate analyses

When exogenous compounds are added to microbial screening, it is critical to first evaluate the antimicrobial properties of these metabolites because, ideally, the amount added to the culture should be at subinhibitory concentrations. Hence, to evaluate the antifungal properties of (–)-cassine and (–)-spectaline in *F. oxysporum*, we have used Disc Diffusion Assay (DDA). This technique was fast and sensitive to determine the degree of inhibition on the microorganism and the inhibition zones were visible within two days of incubation. DDA results revealed that the piperidine alkaloids had a pronounced antifungal potential against *F. oxysporum*, exhibiting inhibition zones of 9.7, 8.7, 7.0, 6.7, and 4.7 ± 1.4 mm at the concentrations of 500, 300, 200, 150, and 100 µg, respectively. The minimum inhibitory concentration was estimated to be between 100–50 µg. For fungi cultures tested with concentrations lower than 50 µg, the growth inhibition was no longer visible, indicating that these compounds had no antibiotic effect over *F. oxysporum* LC055797.1. and could be safely used at the sub-inhibitory level in the feeding experiment.

**Fig. 1:**
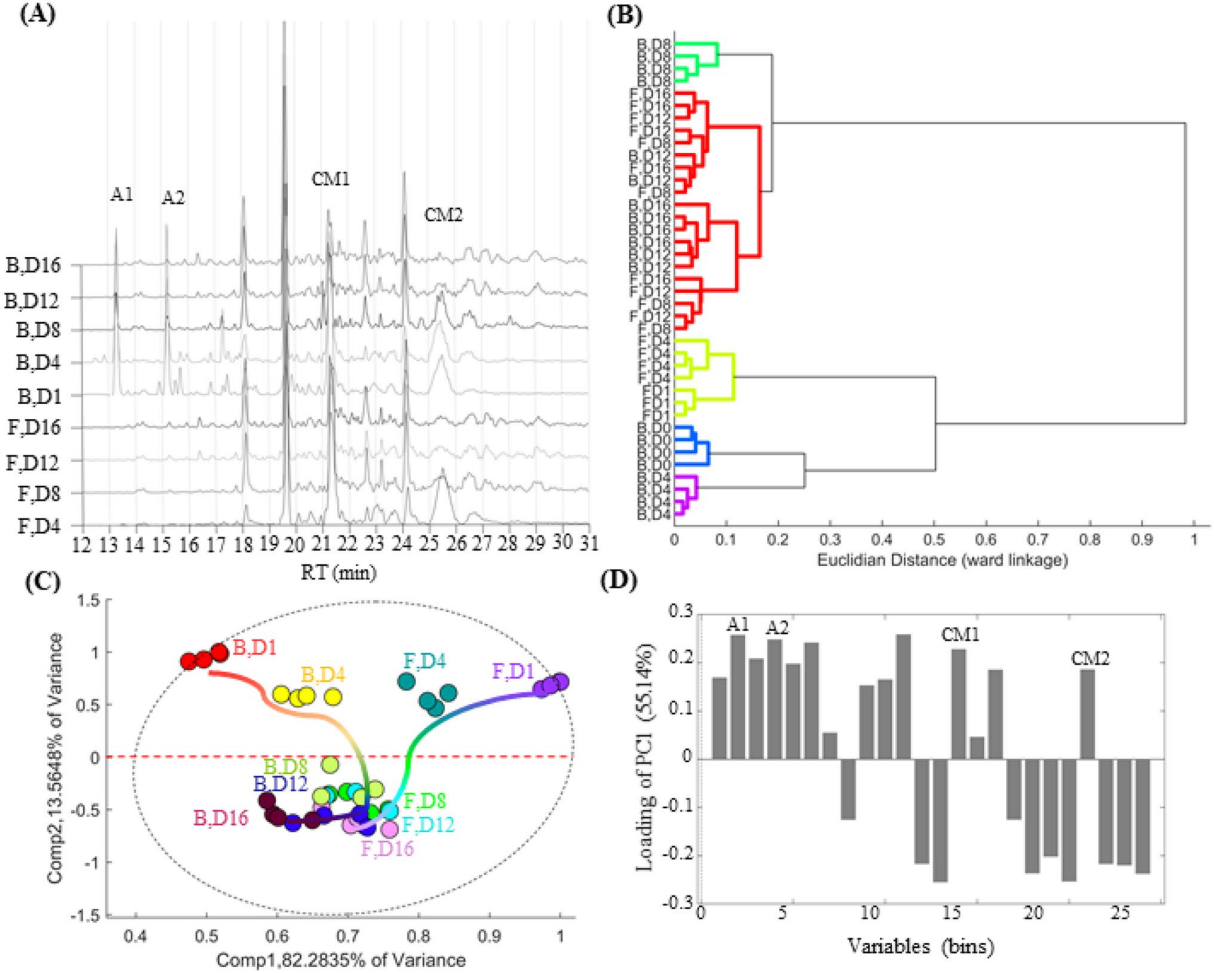
**A** Total ion chromatograms (TIC) of the replicates from the fungal samples with and without alkaloidal addition. Data is displayed as the mean values of the analytical and biological replicates. Samples from the control (without alkaloid) were coded as 'F', and samples with the elicitor were coded as 'B'. The incubation days are represented by the letter D, in which D1, D4, D8, D12 and, D16 represent the raw extract after 1, 4, 8, 12 and, 16 days of fungi growth. **B** HCA- dendrogram of preprocessed and binned TIC data of all replicates. **C** PCA 2D-Score plot of principal component 1 (variance of 83.28%) and PC2 (variance of 13.56%), with a total variance of 95.84%. Samples were majorly discriminated over PC2, with samples from the late fungi growth (days 8,12, and 16) clustered on the negative PC2 values and early growth sampled (days 1 and 4) grouped on the positive PC2 quadrants. **D** PCA 1D-Loading of PC2 shows the chemical differences between samples from the early and late growth stages. Bins that are placed on the negative values of the loading are correlated to the fungi metabolism, whereas the ones that are shown on the positive values are mostly correlated to the culture medium (major culture-medium compounds coded as CM1 and CM2) and the alkaloids (and biotransformed molecules) used in the experiment. Codes A1 and A2 represent cassine and spectaline, respectively)

Initial exploratory analyses of the chemical profiles demonstrated that the concentration of the alkaloids (bins at *t*_R_ 13.3 and 15.4 min for (−)-cassine and (−)-spectaline, respectively) decreases according to the incubation days, suggesting the consumption of these plant-metabolites in the first days of growth (Fig. 1A). Samples of *F. oxysporum* on days 1 and 4 show broader and more intense peaks for these compounds, while on days 8, 12, and 16, these peaks are nearly undetectable. Hierarchical Clustering Analysis (HCA) suggested reproducibility of the measurements, displayed by a small Euclidian distance between the replicates. The fungal samples that grew in the presence of the alkaloids were grouped separately according to the incubation days, demonstrating a significant difference between the profiles of each set (Fig. 1B). This was true for samples on days 1, 4, and 8 and is mainly due to the consumption of the exogenous compounds as well as possible biotransformation and/or metabolic induction caused by the presence of these metabolites. These differences were not observed on samples from days 12 and 16 days, grouping the replicates with and without alkaloidal addition together. This implies that the alkaloids do not cause any permanent alterations in the fungal metabolome and, after its consumption, the chemical profiles are highly similar to the ones without alkaloidal addition. We have also observed that fungal samples on days 1 and 4 without alkaloidal addition were separated from the ones with addition and confirm that the differences observed in the presence of toxic compounds are not from the normal fungal metabolome but due to the alterations caused by these exogenous compounds. Moreover, the clear separation of days 1 and 4 without alkaloidal addition was expected given the changes in metabolic production and media consumption in the early growth stages (exponential growth phase).

PCA revealed an absence of outliers at a 95% confidence level and a total explained variance of 95.84% for PC1 and PC2. Given the common nature of the fungal extract, the first principal component (PC1) contained most of the explained variance (83.28%), followed by the orthogonal PC2, which explains the remaining 13.56% variation from the original data. PCA score plot (Fig. 1C) shows the tendency of discrimination on PC1 and PC2, grouping samples in three distinguished spacial clusters. The first was placed on the positive values of PC2 and lower positive values of PC1 and represented the samples with alkaloidal addition on the early days of incubation (B, D1, and B, D4). The higher distance between those and all the other samples was chemically explained by a higher concentration of both alkaloids, nearly absent in all the other samples from the dataset. The second cluster was also on the positive side of PC2 but in higher values of PC1 and contained the samples without alkaloidal additions on the early days of incubation (F, D1, and F, D4). Those fungal cultures were placed on the same PC2 side as the first group given that they both share a high concentration of media components, as well as low amounts of fungal metabolite. Lastly, the third cluster is the opposite of samples from days 1 and 4 and corresponds to the samples extracted on the later incubation days. Regardless of the addition of the alkaloids, those samples are grouped together on the negative side of PC2, agreeing with HCA and once again suggesting the lack of permanent alterations in the fungal metabolome after alkaloidal addition to the culture media (Fig. 1D). One very important feature of the PCA analysis was the tendency of the samples with and without alkaloidal feeding on the PCA score. As can be seen in Fig. 1D, samples with the piperidine alkaloids formed a U-shaped tendency according to the incubation days, indicating a complete change in the metabolite profile. Contrarily, the control samples showed a linear-shaped vector, suggesting only an increase/decrease in the concentration of its metabolome.

### Chemical fingerprinting of *F. oxysporum* after alkaloidal feeding – supervised partial least square (PLS) analysis

#### Construction of a microbial-specific model

The unsupervised nature of PCA and HCA provides a means to unbiasedly achieve dimensionality reduction. However, its application can only distinguish classes when the within-group variation (replicates) is sufficiently lower than the between-group variation (sample types). This is not the case with most microbial data, which typically generates datasets with similar profiles and strongly correlated variables, requiring supervised forms of analyses that rely on the class membership and modeling of each observation. To achieve that, Partial least squares (PLS) was employed as a supervised modeling approach both as a regression (PLSr) and a classification (PLS-DA) model, giving complementary information about the chemical variation within the profiles.

The most important aspect of a supervised chemometric analysis is the modeling of the data. Mainly, two important factors play a pivotal role in determining the best Y-model for the evaluation of the microbial metabolome according to the incubation days: media availability and the fungi growth rate. This intrinsic relation between metabolome, growth, and nutrients influences both the amount and diversity of the metabolic production and, as such, directly interferes in the statistical evaluation and comparison of their metabolome. For instance, if the microbe grows fast, the consumption of the culture media will be faster, decreasing nutrient availability and increasing abiotic stress in the early stages of incubation. This nutrient privation is particularly relevant for microbial metabolomics because stress can lead to the induction of secondary metabolites and the consumption of other specialized compounds. Contrarily, if the microbe has a slow growth rate, or if the strains are exposed to some early source of stress that slows their growth (such as the presence of toxic exogenous compounds), we expect more intense culture media components in the late growth stages and slower production of the secondary metabolites, also hindering the comparison and statistical data analysis. Regardless of the case, in most experiments, the metabolic production in microbes does not happen at the same rate. Hence, using incubation days as the only criteria for data modeling would fail to accurately predict the metabolic outcome, hampering the comprehensive understanding of the microbial metabolome.

It is also abundantly reported that culture media availability in a Petri dish leads to a population growth that follows a logarithmic scale, displaying three distinct phases: lag phase, exponential growth phase, and stationary phase. Hence, it would stand to reason that the metabolomic production would also follow the same tendency, correlating the metabolome to the amount of nutrients available for fungal development. In fact, a standard log-transform on the initial linear vector was successful in increasing both the predictive ability of the model and the statistical significance for both datasets (feeding and without feeding), exhibiting higher R2 on cross-validation and lower p-value than the analysis that used incubation time without treatment (Table 1).

**Table 1:** Selection of the best model for the supervised chemometric analysis using microbial-specific parameters

	Fungi with alkaloidal feeding				Fungi without feeding			
	Incubation days		Diameter of the colony		Incubation days		Diameter of the colony	
	Model with no treatment	Model after log- treatment	Model with no treatment	Model after log- treatment	Model with no treatment	Model after log- treatment	Model with no treatment	Model after log- treatment
Class 1	1	0	1	0	1	0	1	0
Class 2	4	1.38	3.19	1.16	4	1.38	4.9	1.58
Class 3	8	2.07	7.54	2.02	8	2.07	8	2.07
Class 4	12	2.48	8.41	2.12	12	2.48	8.5	2.14
Class 5	16	2.77	9	2.19	16	2.77	9	2.19
R2 CV	0.9734	0.9861	0.9852	0.9889	0.9205	0.9294	0.9772	0.9843
p-val Wilcoxon	0.0001*	0.0001	0.0001	0.0001	0.003	0.003	0.001	0.0001
p-val Sign test	0.002*	0.0001	0.0001	0.0001	0.021	0.028	0.006	0.002
p-val t-test	0.005*	0.005	0.005	0.0001	0.009	0.007	0.005	0.005

The choice was based on the difference in the PLSr validation parameters. The best model is the one that shows the higher R2 value on cross-validation and the lowest p-values on the permutation test. The columns are first divided according to the dataset: the first three columns are from *Fusarium* samples with alkaloidal feeding, and the last three columns are from the fungal samples without feeding (control). Rows 1–5 represent the numerical values of the Y-vectors according to the treatment performed. The last rows indicate the R2 values of the cross-validation and the empirical p-values of the permutation test with 400 iterations. The permutation test was applied using Wilcoxon, Sign test, and t-test for self-prediction (SP) and cross-validation (CV), and the values displayed in the Table correspond to the highest between SP and CV. Pictures of the Petri-dish containing the fungi colony on each incubation day and the results of the different treatments in the Y-vectors are available in Figure SM5 of the Supplementary Material

Although the initial treatment of the Y-vector improved the quality of the PLSr analysis, the use of log-transformation on the vector of incubation days failed to account for the differences in the growth rate when comparing the chemical profile of two microbial types. These growth differences are significantly misleading when comparing two species with different growth rates or even one strain evaluated under other conditions. In both cases, metabolic variation happens in different time frames, hampering variable correlations and chemical interpretation. To date, no correlation between these parameters for the modeling of supervised methods has been reported in the literature.

To account for both factors described above, we have created a simple approach based on the fact that the colony size and growth rate could be visually assessed in solid media. Moreover, the use of logarithmic transformation of the colony sizes corrects the influence that both media availability and growth differences have on metabolic production. To account for fungi growth rate, we used the measurement of colony diameters (in cm) in Petri dishes on each incubation day. Following, these values were submitted to a log transformation to consider media availability, resulting in a transformed Y-vector for data modeling. When compared to the other Y-vector models, this approach showed the highest predictive ability and statistical significance (R2 CV 0.9889 and p-value 0.0001), encouraging their use for any microbial culture grown in solid media (Table 1).

#### Linear chemical variation in different incubation periods—PLS regression (PLSr)

We hypothesized that the metabolites either consumed or produced according to the fungi growth would be shown as linear regression. This is the case of metabolites from the culture media and metabolites from fungus metabolism. Both metabolic groups are correlated (negatively and positively, respectively) to the incubation time and dictate essential aspects of the fungus metabolism and carbon source consumption. The PLSr analysis was performed separately for both datasets to provide comparative patterns of the metabolic variation of *F. oxysporum* with and without alkaloidal feeding. The main goal of this MDV analysis was to use the model to (1) identify new metabolites that are produced over time, as well as to (2) verify the possible induction/suppression of known *Fusarium* secondary metabolites due to the presence of the exogenous alkaloids. The identification of these compounds was performed by a comparative analysis of PLSr results in both datasets. The bins from the same RT were evaluated regarding their UV-DAD, HRMS, and tandem MS profiles. Only bins that have a similarity rate of over 95% in all three profiles were considered the same. The metabolites that have a statistically significant correlation in the regression were selected by the Variable Influence on Projection (VIP). Only VIPs bigger than a certain threshold (in our case, VIPs > 1.00) are considered for analysis, and the higher the VIP value is, the greater the correlation of this bin (variable) to the model.

**Fig. 2:**
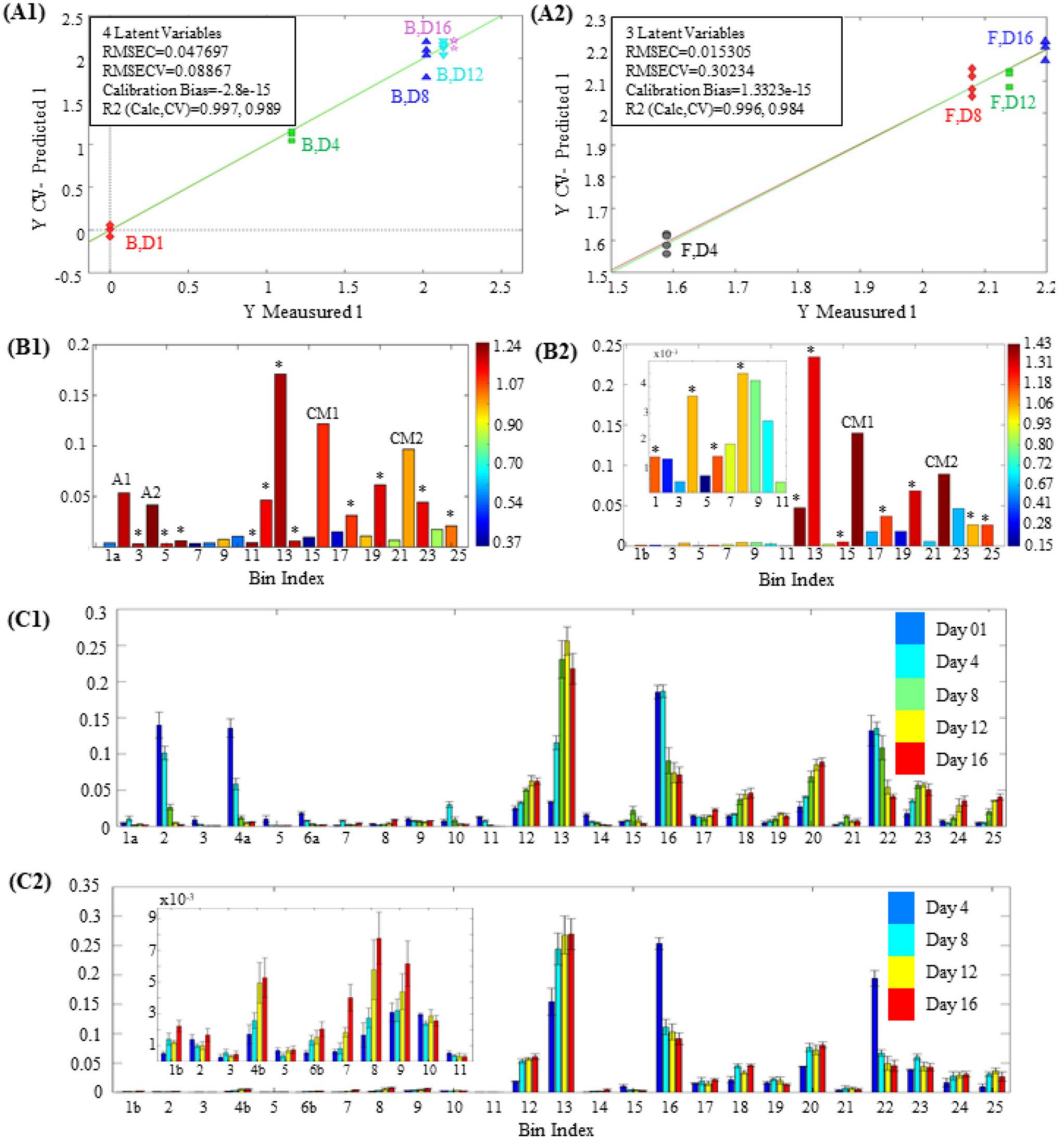
**A** Correlation between measured and predicted output for *F. oxysporum* with (A1) and without (A2) alkaloidal addition, both derived from the microbial-specific PLSr models using the bins of TIC data. Analyses were validated by cross-validation and permutation tests. **B** The total area of each bin from *F. oxysporum* with (B1) and without (B2) alkaloidal feeding was colored according to the VIP scoring from PLSr analysis. The threshold for the selection of appropriate bins was VIPs > 1.00. The statistically relevant bins (VIP > 1.00) are colored in dark red and signalized with (*). **C** Mean values and standard deviation of the total area for each bin from *F. oxysporum* with (C1) and without (C2) alkaloidal feeding, plotted according to the incubation day. Bins that have the same number but different letters (e.g., 1a and 1b) between plots C1 and C2 correspond to bins that contain elute in the same retention time but show different mass spectra profiles and are, therefore, considered to be different metabolites

For both data types, PLSr revealed an absence of outliers and predictive values that were in close agreement with the actual experimental data, exhibiting R2 values from cross-validation of 0.989 and 0.984 for the samples with and without alkaloids, respectively (Fig. 2A). Overall, the metabolites that are statistically relevant in the PLSr with exogenous compounds are also significant to the PLSr analysis of the control samples. These results mean that regardless of the alkaloidal feeding, those metabolites are positively (if produced) or negatively (if consumed) correlated to the incubation days (Fig. 2B and C). This is the case for bins 12, 13, 16, 18, 20, 22, and 25 and supports our initial conclusion that these profiles have high similarity (identity and concentration) and that the presence of these exogenous compounds does not have a permanent impact on fungus metabolic production. Furthermore, bins 2 and 4, attributed to the piperidine alkaloids (coded as A1 and A2), follow a negative regression on samples with feeding, also proving that these compounds are biotransformed or degraded by the fungi over time, being nearly undetected after day 12 of incubation.

A more thorough analysis of the significant variables was performed using the total area values according to the incubation days (Figure SM8). It showed that from the 25 bins in which the TIC data was initially divided, 15 and 13 were selected as statistically relevant in the PLSr analyses for samples with and without alkaloidal addition, respectively. Seven bins were significant for both data types and represented the culture media components and the fungal metabolism under standard monoculture conditions. Contrarily, eight were considered important only to the samples with alkaloidal feeding and indicated the chemical variation due to the presence of these exogenous compounds. Out of those, seven showed negative correlations and included the alkaloids (-)-cassine and (-)-spectaline (bins 2 and 4a) and minor contaminants from the alkaloidal purification process (bins 3, 5, 6a, 11, and 14). Only bin 23 displayed a positive correlation, produced in low but increasing abundance according to the incubation days. Lastly, although there were five bins correlated exclusively to the *Fusarium* control (bins 1b, 4b, 6b, 15, 24), manual inspection of the MS and UV data showed that these compounds are also present in the fungal samples with alkaloidal feeding. However, in the latter, they appeared convoluted with more abundant peaks, hampering their identification and correlation.

#### Classification of metabolites in different incubation periods—PLS discriminant analysis (PLS-DA)

Notwithstanding the advantages of PLSr, this technique alone could not shed light on the general metabolic variation during the microbial life cycle. This limitation happens because in metabolomics studies multiple and potentially interacting factors influence the data. Very often, the production/consumption of a molecule cannot be considered a pure regression. For instance, several metabolites are only produced at a specific period of growth in response to the presence of a competitive organism or, in our case, of a toxic compound. In those cases, PLS-DA would be capable of identifying the production of other metabolites that do not follow regression but are equally important for the biological and ecological understanding of these strains.

In this study, PLS-DA was applied to evaluate the differences in the metabolic production on each day, discriminating distinct sets of metabolites from each period of the fungus growth. Chemically speaking, PLS-DA aimed to correlate the metabolites that were temporarily produced (i.e., produced and consumed in a short period) as an immediate reaction to abiotic and biotic stress, inferring information of specific metabolic modulation in these microbial matrixes. Initial PLS-DA using the treated Y-vector as a model showed a clear separation between samples from days 1, 4, and 16. Nonetheless, this model was unable to differentiate between days 8 and 12. Indeed, the assessment of different Y-class models based on the growth (e.g., early/late growth stage, lag/log/stationary growth phase) failed in cross-validation and permutation tests. Only the Y-model that groups samples from days 8 and 12 into one single class (1, 4, [8/12], 16) showed > 95% confidence and could be used to infer chemical variation.

**Fig. 3:**
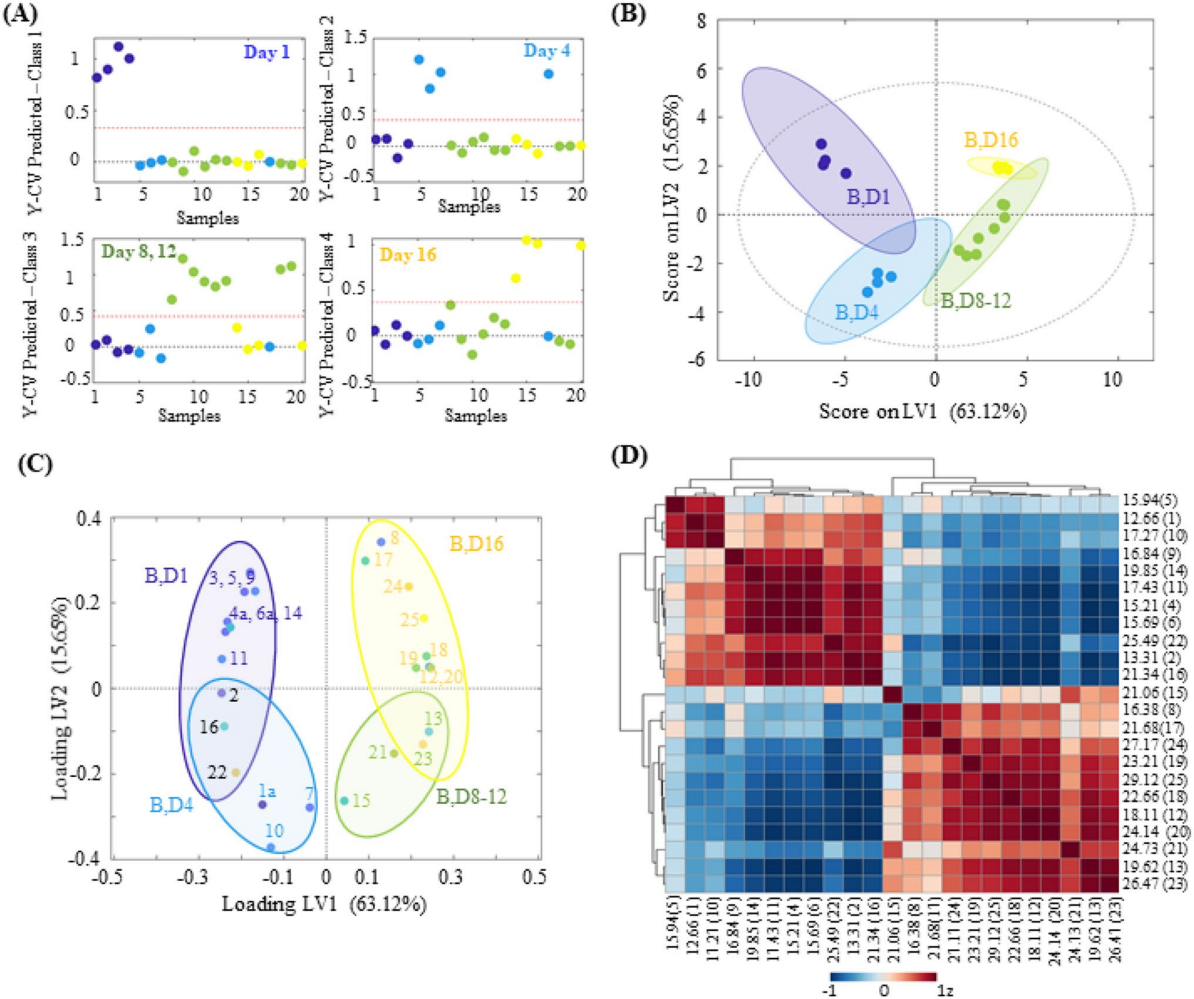
PLS-DA was built to investigate the effects of alkaloidal feeding and check the metabolites produced temporarily as a response to the presence of the exogenous compounds. **A** PLS-DA classification, according to the incubation days. The red dashed lines indicate the classification boundary. Samples belonging to the targeted class are located on the top, while samples belonging to the other two sample groups are located at the bottom. **B** Score scatter plot of PC1 and PC2 (total explained variance of 78.77%), where each class is represented in a colored circle that represents a 95%-confidence level. Samples were discriminantly placed in the quadrant of the score plot, with samples from day 1 on PC1-PC2 + , samples from day 4 on PC1-PC2-, samples from days 8/12 on PC1 + PC2- and samples from day 16 on PC1 + PC2 + . **C** 2D-Loading scatter plot of the PC1 and PC2. The variables (circles) are reported in the same plot quadrants as the correlated samples from the score plot. **D** Heat maps of all 25 bins. Data of the relative concentration of bins were analyzed using Spearman rank correlation analysis from MetaboAnalyst (v 4.0). Each colored cell on the map indicates the correlation coefficient, with the scale code shown in the top right corner. The red-like color indicates positive correlations, while blue-like colors mean negative correlations. Each bin is displayed on the heatmap according to its retention time (in minutes), followed by the bin number in parenthesis

The “goodness” of the PLS-DA model, measured by R2 and Q2, showed that no over-fitting was observed and, consequently, this model was successful for the discernment according to the 4-class model (Fig. 3A). The calibration results used eight principal components which sum up to 97.85% of the explained variance, 78.77% of which are within PC1 and PC2. The score for the dataset can be seen in Fig. 3B. Each class was placed in one quadrant of the 2D-score plot between PC1 and PC2, distinguishing days 1 (PC1-, PC2 +), day 4 (PC1-, PC2-), days 8/12 (PC1 + , PC2-), and day 16 (PC1 + , PC2 +). Just as previously observed on the unsupervised PCA, the same U-shaped tendency was also formed in PLS-DA, once again highlighting the drastic change in the metabolic profile according to the incubation period represented by the increase in fungal metabolome and decrease of the piperidine alkaloids and culture media compounds.

Evaluation of the loading values from PCs 1–2, displayed in Fig. 3C, revealed the chemical differences between classes and the success of the PLS-DA in discriminating metabolites according to the incubation days. This excellent separation was not observed on the *Fusarium* control data, in which all the PLS-DA attempts using many different models always resulted in overfitting and lack of validation. As a general rule, variables at the extreme corners of the loading plot contribute the most to the separation, and for each class, only metabolites with VIP values bigger than one were considered significant. All the peaks that were considered relevant on the PLSr analysis were attributed to either class 1 (i.e., day 1, when the correlation was negative) or class 4 (i.e., day 16, when the correlation is positive). This behavior was expected because these bins follow a linear regression and hence, have their highest intensity at either the beginning (culture media metabolites) or the end (fungus metabolism) of the incubation period. That was the case for the bins with negative correlations 2, 3, 4, 5, 6, 9, 11, 14, 16, 22, and positive correlations 8, 12, 13, 17, 18, 19, 20, 23, 24, 25, the details of each were described above that will not be further analyzed here (Fig. 3D).

The most exciting results arose from the chemical analysis of classes 2 and 3, which represent days 4 and 8/12 of incubation, respectively. In those, almost all metabolites associated with the PLS-DA separation were not considered statistically significant on the PLSr, suggesting that those were temporarily produced as a response to the presence of the piperidine alkaloids. Evaluation of the chemical profiles of the bins selected for these classes showed that, except for bin 13 (be, all the metabolites were exclusively produced only in the presence of the plant metabolites, while also being absent in all the LC–MS data from the control group.

The PLSr analysis of the samples with elicitor molecules revealed that the alkaloids (bins 2 and 4) were majorly consumed between days 1 and 8. This piece of information was relevant to the analysis of PLS-DA loadings because, during this same period, there was a significant increase in the production of compounds from different polarities that were not produced in standard conditions. This tendency can be easily seen by the heatmap plot from Fig. 3D, emerging the hypothesis of a specific chemical variation in the presence of the exogenous compounds. For class 2, bins 1, 7, and 10 were exclusively attributed to day 4 of incubation and represented mostly polar metabolites that were produced (by induction of BCG or biotransformation of the alkaloidal compounds) as a first response to the toxicity stress. For class 3, on the other hand, the production of two apolar metabolites (bins 15 and 21) was attributed as a late response to the alkaloidal feeding, being expressed exclusively between this period.

Furthermore, for samples from the incubation days between 8 and 12, there was also a pronounced increase in intensity of bin 13. This bin (*m/z* 806.3995 [M + Na]^+^ and *m/z* 784.4146 [M + H]^+^; *t*_*R*_ 19.85 min) corresponds to a known *Fusarium* metabolite named beauvericin (CAS number 26048–05-5), which is a depsipeptide encountered in many strains of *Fusarium* and already reported for this strain of *F. oxysporum* in previous works (Selegato et al., 2016)*.* Although this mycotoxin was produced regardless of the presence of the alkaloids, its production was increased in the presence of (–)-cassine and (–)-spectaline, showing positive modulation during stress.

### Differential metabolomic annotation

The combined analysis of PLSr and PLS-DA has inferred important chemical features of the microbial data, enabling a comprehensive evaluation of the fungal metabolite. An “induced” metabolite was defined by the satisfaction of three criteria: high VIPs values in PLSr or PLS-DA, p-value < 0.005 from t-test that compares the peak area on both datatypes (suggesting a statistical difference between the two groups), and a mean peak area at least threefold higher in the treatment than the control. On the one hand, PLSr revealed that bin 23 was exclusively produced in the presence of the alkaloids, being induced as a linear regression according to the incubation day. On the other, PLS-DA could identify polar and apolar compounds (bins 1, 7, 10, 15, 21) that were temporarily produced in the presence of the exogenous compounds, shedding light on the early and late chemical responses of the fungi.

Preliminary analysis of the selected bins was based on the evaluation of the most abundant member(s) by the combined analysis of UV and MS data (putative metabolite annotation). Molecules were considered to belong to the same metabolite class whenever they shared the same UV absorption and tandem mass spectra, as well as similar height proportions between the [M + H]^+^ and the [M + Na]^+^ ions. These compounds are detailed in Table 2. Further analytical description of these molecules is also available in Figure SM12 from the Supplementary Material.

**Table 2:** Experimental data of the metabolites significant statistically for the PLS analysis

Bin n	Retention time (min)	*m/z* experimental	Molecular Formula	*m/z* theoretical	Error (ppm)
1	12.66	448.4034	C_18_H_35_N_2_O	448.4043	2
2	13.31	298.2761	C_18_H_35_NO_2_	298.2746	5
4	15.21	326.3051	C_20_H_39_NO_2_	326.3059	2.4
7	15.94	328.3230	C_20_H_41_NO_2_	328.3215	4.5
10	17.27	344.3177	C_20_H_41_NO_3_	344.3164	3.7
13	19.62	784.4146	C_45_H_57_N_3_O_9_	784.4170	3.0
15	21.06	599.4111	C_25_H_51_N_12_O_5_	599.4105	0.94
21	24.73	639.5315	C_37_H_71_N_2_O_6_	639.5312	0.45
23	26.47	617.5467	C_38_H_71_N_3_O_3_	617.5495	4.6

Bins number 1 (*m/z* 286.2668 and minor ion at *m/z* 448; *t*_R_ 12.65 min), n. 7 (*m/z* 328.3215; *t*_R_ 15.94 min), and n. 10 (*m/z* 344.3164 and ion at *m/z* 583; *t*_R_ 17.27 min), detected mainly on day 4 of incubation, shared the same UV and MS patterns as (–)-cassine (bin 2, *m/z* 298.2746; *t*_R_ 13.31 min) and (–)-spectaline (bin 4, *m/z* 326.3059; *t*_R_ 15.21 min). Therefore, these were classified as analogues of the targeted alkaloids. This result indicates that *F. oxysporum* promptly biotransforms the alkaloids into analogues as a defense mechanism to evade toxicity. All these bins had the same maximum UV − visible absorption at 200 nm and displayed a similar proportion between the proton and sodium adducts peaks (greater than 1:40). The accuracy of the high-resolution masses, together with the alkaloidal structures, suggested common chemical modifications that could be deduced from decreased or increased masses of the piperidine alkaloids. For instance, the positive mass differences of + 2.016 (from bin 4 m*/z* 328 to bin 7 m*/z* 326) and + 18 (from bin 4 m*/z* 326 to bin 10 m*/z* 344) suggest hydrogenation (+ H_2_) and hydration (+ H_2_O) of (-)-spectaline, respectively.

The second group of metabolites, whose production was only observed in the presence of the alkaloids after 8 and 12 days of incubation is bins 15 (*m/z* 599.4111; *t*_R_ 21.06 min), 21 (*m/z* 639.5315; *t*_R_ 24.73 min), and 23 (*m/z* 617.5467; *t*_R_ 26.47 min). These ions are compatible with highly polar compounds that were almost undetected in the control samples. However, those were not identified using Metlin, MoNA, and MassBank (Horai et al., 2010) databases.

Lastly, a comparative antimicrobial assessment was performed between (1) the extracts of fungi with alkaloidal addition (B1, B4, B8, B12, and B16) and (2) the fungal extracts as control (F4, F8, F12, and F16). These analyses were performed against Gram-positive strains (*Bacillus cereus* and *Staphylococcus aureus*) and two gram-negative strains (*Pseudomonas fluorescens* and *Escherichia coli)*. For gram-positive microbes, the samples with higher concentrations of the piperidine alkaloids showed high antimicrobial activity against *S. aureus* and *B. cereus* (MIC of 128 µg.mL^–1^), whereas after alkaloidal incubation, the antimicrobial activity is absent. This indicates that the presence of this fungi species reduces the antimicrobial effect of the piperidine alkaloids by biotransformation, producing metabolites that are either inactive against these strains or present in a subinhibitory concentration on the fungal extracts.

## Conclusion

Relatively little is known about the activation of silent BGCs by the presence of elicitors or the changes of abiotic features. In this sense, the use of wild microbes and molecules present in high abundance in their natural environment can shed light on the ecological and biological evaluation of the strains. Moreover, in the case that these exogenous molecules are biologically meaningful, the feeding of microbial strains may also lead to potential analogues that are difficult to obtain synthetically, as well as induced bioactive secondary metabolites, resulting in unlimited possibilities for chemical enhancement on different microbial strains.

Statistical pipelines that allow a comprehensive analysis of the metabolome in microbial cultures are also neglected in current metabolomics literature. In this sense, the treatment of the Y-data and the combined use of PLS-DA and PLSr has inferred important and complementary chemical features of the microbial data. On the one hand, PLSr has revealed the metabolites that are consumed or produced according to the fungi growth. This is the case of metabolites from the culture media and metabolites from the fungus metabolism, both metabolic groups that are correlated to the incubation time. On the other hand, PLS-DA has focused on metabolites that are only consumed/produced at a specific period, in response, for example, to specific environmental variation. Both classificatory and regression analysis are equally crucial for the biological and ecological understanding of these strain and enables the monitoring of the microbial metabolome and the identification of compounds that are only temporarily produced by the strains.

Although the metabolites produced by *F. oxysporum* in the presence of the piperidine alkaloids remain to be identified, the results indicate that our approach can efficiently detect and correlate metabolites variation according to microbial growth. In fact, in microbial metabolomics, these supervised techniques have been increasingly applied to investigate the relations between environmental factors, phenotypes, and metabolism. Notwithstanding its advantages, it is important to keep in mind that these methods might provide misleading results when used by non-experts who are not aware of their potential limitations. The main challenges include bias, inadequate sample size, excessive false discovery rate, wrong choice of chemometric technique, and overfitting, failing to discover anything of real significance. Too many studies do not assess model performance, wrongly concluding that the job is done after obtaining a good separation on the score plot. Lastly, it is also important to keep in mind that PLS methods are often susceptible to over-fitting because they may produce patterns even in random data. Therefore, cross-validation and permutation tests are mandatory steps in the MDVA workflow and should receive more attention in both targeted and untargeted metabolomics analysis.

The major drawback of microbial metabolomics is the fact that the production of specialized metabolites is often sensitive to external factors, hampering reproducibility and the identification of minor metabolites. Hence, for a reliable evaluation of specialized metabolites, it is imperative to culture the strains under identical abiotic and biotic conditions, as well as use sensitive analytical techniques. Other measures to correct these challenges also include the use of appropriate control (isolated strains and only culture medium) and the acquisition of several biological/technical replicates. All these precautions were taken in this study and have improved the sensitivity and precision of both PLS regression and discrimination.

## Supplementary Information

Below is the link to the electronic supplementary material.

Supplementary file1 (PDF 2616 kb)
