# Supplementary material for: Time-related multivariate strategy for the comprehensive evaluation of microbial chemical data
Source: Metabolomics. 2022 May 24;18(6):33. doi: 10.1007/s11306-022-01896-6 (PMC9130195; doi:10.1007/s11306-022-01896-6)
Supplement: Supplementary file 1 — Supplementary file1 (PDF 2616 kb) [file 11306_2022_1896_MOESM1_ESM.pdf]

SUPPLEMENTARY MATERIAL

Time-related Multivariate Strategy for the Comprehensive Evaluation of Microbial Chemical Data

Chemical Response of *Fusarium oxysporum* to Toxic Plant-metabolites

Denise M. Selegato[1,ψ,*], Thamires R. Freitas[2], Marcos Pivatto[2], Amanda D. Pivatto[2], Alan C. Pilon[3], Ian Castro-Gamboa[1]

[1] Nucleus of Bioassays, Biosynthesis, and Ecophysiology of Natural Products (NuBBE), Institute of Chemistry, São Paulo State University, Araraquara, SP, Brazil.
[2] Núcleo de Pesquisa em Compostos Bioativos (NPCBio), Instituto de Química, Universidade Federal de Uberlândia, Uberlândia, MG, Brazil.
[3] Núcleo de Pesquisa em Produtos Naturais e Sintéticos (NPPNS), Faculdade de Ciências Farmacêuticas de Ribeirão Preto, Universidade de São Paulo, Ribeirão Preto,SP, Brazil.

[ψ]Current address: Zimmermann Laboratory, Structural and Computational Biology Unit, European Molecular Biology Laboratory (EMBL), Heidelberg, Germany.

*Corresponding author: Dr. Denise M. Selegato; e-mail: denisemselegato@gmail.com; telephone +49 1733857809.

Fusarium oxysporum genetic identification

F. oxysporum was isolated from the rhizosphere of *S. spectabilis* in the Southeast region of Brazil in July of 2012, one of the most comprehensively studied sites in Latin America, with a microbial community dominated by *Fusarium*. The genetic identification was done by the analysis of the Internal Transcribed Spacer (ITS) sequence and deposited at GenBank under access number LC055797.1.

Isolation of Targeted Piperidine Alkaloids from _S. spectabilis_ – extraction and isolation

S. spectabilis flowers were collected by Dr. Pivatto in Araraquara (São Paulo, Brazil) in July 2010. The plant was identified by Dr. Inês Cordeiro from Instituto de Botânica in São Paulo-SP, Brazil. A voucher specimen (SP 384109) was deposited in the herbarium of this Institute.

S. spectabilis flowers (38.0 kg) were dried in an air-circulation oven for five days. The dried material (7.9 kg) was powdered with a knife mill and subjected to extraction with 5 liters of ethanol HPLC-grade (EtOH) at room temperature for seven days. The resulting organic extract was then filtered in a qualitative Qualy filter paper 80 $g.m^{-2}$, and the solvent was removed under reduced pressure in a rotary evaporator. This procedure was repeated five times providing a final 1.9 kg of thick syrup. The crude ethanol extract (100.0 g) was dissolved in 500.0 mL of $MeOH/H_2O$ (4:1), filtered and submitted to a sequential liquid-liquid extraction with *n*-hexane (250 mL × 5, 2.6 g) and $CHCl_3$ (250 mL, × 5, 40.5 g), respectively. The remaining aqueous fraction (23.1 g), and all the phases were concentrated under reduced pressure and analyzed by thin-layer chromatography (TLC) on silica gel F_{254} plates; $CHCl_3/MeOH/NH_4OH$ (9:1:0.25) was used as developing solvent system. Spots were visualized with IClPt, to confirm the presence of alkaloids. The alkaloids were concentrated in the $CHCl_3$ fraction (10.8 g) which was submitted to CC according to reported methodology (Freitas et al. 2018). This procedure furnished the pure alkaloids (–)-cassine (**1**) (t_R 17.4 min, 1.5 g) and (–)-spectaline (**2**) (t_R 19.7 min, 100.0 mg), which were analyzed by GC-MS (Figure SM1, Supporting Information). The compounds (-)-cassine and (-)-spectaline have CAS numbers of 5227-24-7 and 65560-25-0, respectively.

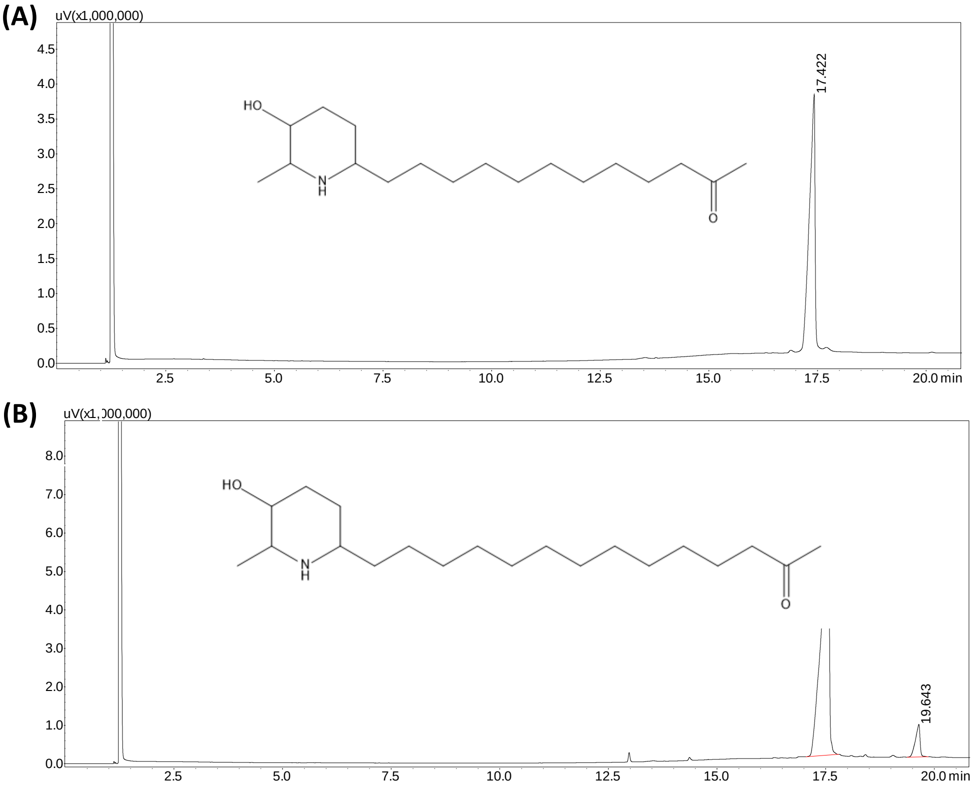

(C)

Peak#	Ret.Time	Area	Height	Conc.	Area%	Height%	Initial Time	Final Time
1	17.569	76021947.2	5644880.0	92.43533	92.4353	87.0226	17.052	17.785
2	19.643	6221440.5	841805.1	7.56467	7.5647	12.9774	19.377	19.848

Figure SM1. CG-MS Analysis of **(A)** (–)-cassine (t_R 17,422 min), **(B)** (–)-spectaline (t_R 19,672 min) and **(C)** Isolated alkaloidal mixture cassine/spectaline (92% C, 8% S).

Concentration Alkaloid (µg) in the well	Inhibition zone diameter (mm)		
	**Piperidine Alkaloids**	**Fungal extracts without feedings**	**Fungal extracts after alkaloidal addition**
500	9.7 ± 1.4	N/D	N/D
300	8.7 ± 1.4	N/D	N/D
200	7.0	N/D	N/D
150	6.7 ± 1,4	N/D	N/D
100	4.7 ± 1.4	N/D	N/D
50	0	N/D	N/D

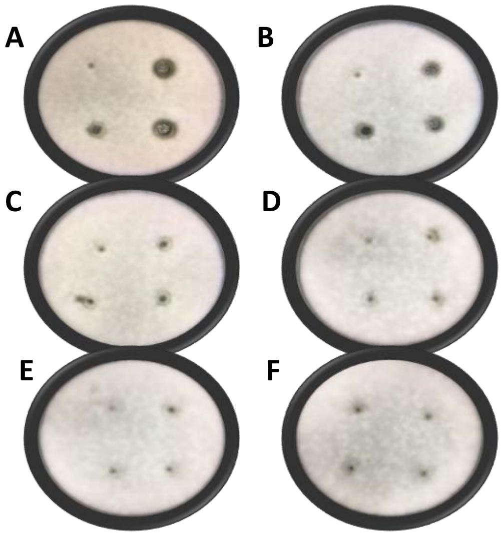

Figure SM2. The selection of the specialized metabolite added to the fungal culture and their optimum concentration are based on their solubility on the solid media, biological activity, and toxicity towards the selected strain. Hence, for the selection of an optimum concentration of the piperidine alkaloids, *F. oxysporum* was submitted to a disc diffusion assay using different alkaloidal concentrations. **Table:** Positive results show an inhibition zone > 0 after nine days of incubation. The diameter of the inhibition zone (area with no apparent growth around the application point) is directly proportional to the antifungal potential of the molecules. The bigger the values, the higher the antifungal capacity. Each assay was measured in triplicates, and the displayed values refer to the mean value ± standard deviation. N/D corresponds to a lack of inhibition zone. **Images of the Disc Diffusion Assay:** using **(A)** 500 µg, **(B)** 300 µg, **(C)** 200 µg, **(D)** 150 µg, **(E)** 100 µg, (F) 50 µg of piperidine alkaloids. Positive results show an inhibition zone > 0 after nine days of incubation. The diameter of the inhibition zone (area with no apparent growth around the application point) is directly proportional to the antifungal potential of the molecules. The bigger the values, the higher the antifungal capacity. Each assay was measured in triplicates, and the displayed values refer to the mean value ± standard deviation. N/D corresponds to a lack of inhibition zone.

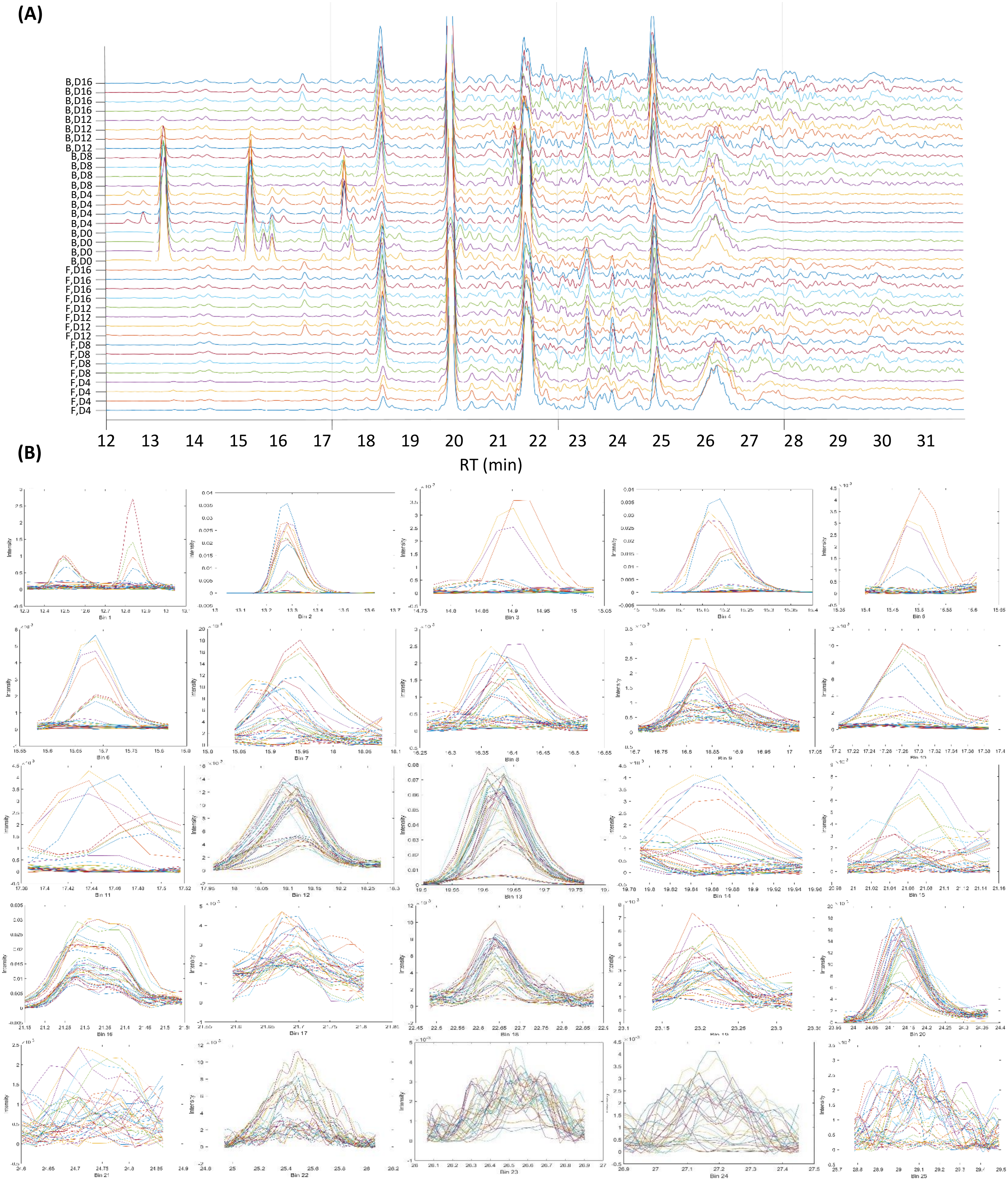
(A)
B,D16
B,D16
B,D16
B,D16
B,D12
B,D12
B,D12
B,D12
B,D8
B,D8
B,D8
B,D8
B,D4
B,D4
B,D4
B,D4
B,D0
B,D0
B,D0
B,D0
F,D16
F,D16
F,D16
F,D16
F,D12
F,D12
F,D12
F,D12
F,D8
F,D8
F,D8
F,D8
F,D4
F,D4
F,D4
F,D4
12 13 14 15 16 17 18 19 20 21 22 23 24 25 26 27 28 29 30 31
RT (min)
(B)

(C)

Bin	Retention time (min)	f.value	p.value	-log10(p)	FDR
1	12.665	20.845	5.50E-06	5.2595	6.66E-06
2	13.31	363.46	9.86E-15	14.006	1.13E-13
3	14.9	143.91	9.80E-14	13.022	1.04E-13
4	15.21	371.19	8.44E-15	14.074	1.13E-13
5	15.49	133.18	4.30E-14	13.882	1.56E-13
6	15.69	165.18	3.30E-12	11.482	1.52E-11
7	15.94	55.333	8.39E-09	8.0764	1.48E-08
8	16.38	44.736	3.65E-08	7.4381	5.99E-08
9	16.84	15.17	3.75E-05	4.4257	3.75E-05
10	17.275	83.376	4.64E-10	9.3333	1.19E-09
11	17.435	177.37	1.96E-12	11.708	1.50E-11
12	18.115	73.861	1.10E-09	8.9587	2.53E-09
13	19.62	131.3	1.76E-11	10.755	6.74E-11
14	19.8525	56.208	7.52E-09	8.1239	1.44E-08
15	21.06	22.736	3.19E-06	5.4959	4.08E-06
16	21.345	92.03	2.29E-10	9.6402	6.58E-10
17	21.685	17.785	1.46E-05	4.8364	1.60E-05
18	22.665	34.133	2.27E-07	6.6434	3.27E-07
19	23.215	15.187	3.73E-05	4.4287	3.75E-05
20	24.145	107.26	7.61E-11	10.119	2.50E-10
21	24.73	19.338	8.75E-06	5.058	1.01E-05
22	25.49	60.028	4.75E-09	8.3235	9.93E-09
23	26.47	41.706	5.89E-08	7.2301	9.03E-08
24	27.175	26.503	1.20E-06	5.9214	1.62E-06
25	29.125	166	3.18E-12	11.498	1.52E-11

Figure SM3. (A) Total Ion Chromatogram (TIC) of the fungi with alkaloidal feeding (coded as B) and the control without the addition of exogenous molecules (coded as F) at different incubation periods. Reverse-phase LC separation was performed for samples extracted at days 1, 4, 8, 12, and 16 of fungal growth. The final matrix represents the intensity of each TIC. The columns contain 25 asymmetric bins (reduced from 745 data points) and the rows 36 sample entries (three replicates for each sample plus the mean value for each class). **(B)** The final matrix was preprocessed and binned, totaling 25 regions that represent the most abundant signals from the TIC chromatogram. Each plot represents the stacked plot of all samples for each bin. Color is random. **(C)** p-Values from Mixed Analysis of Variance (ANOVA) test of each bin. Only statistically significant bins were used. Analysis was performed at the MetabolAnalyst website, available at: https://www.metaboanalyst.ca/MetaboAnalyst/ModuleView.xhtml. FDR represents False Discovery Rate.

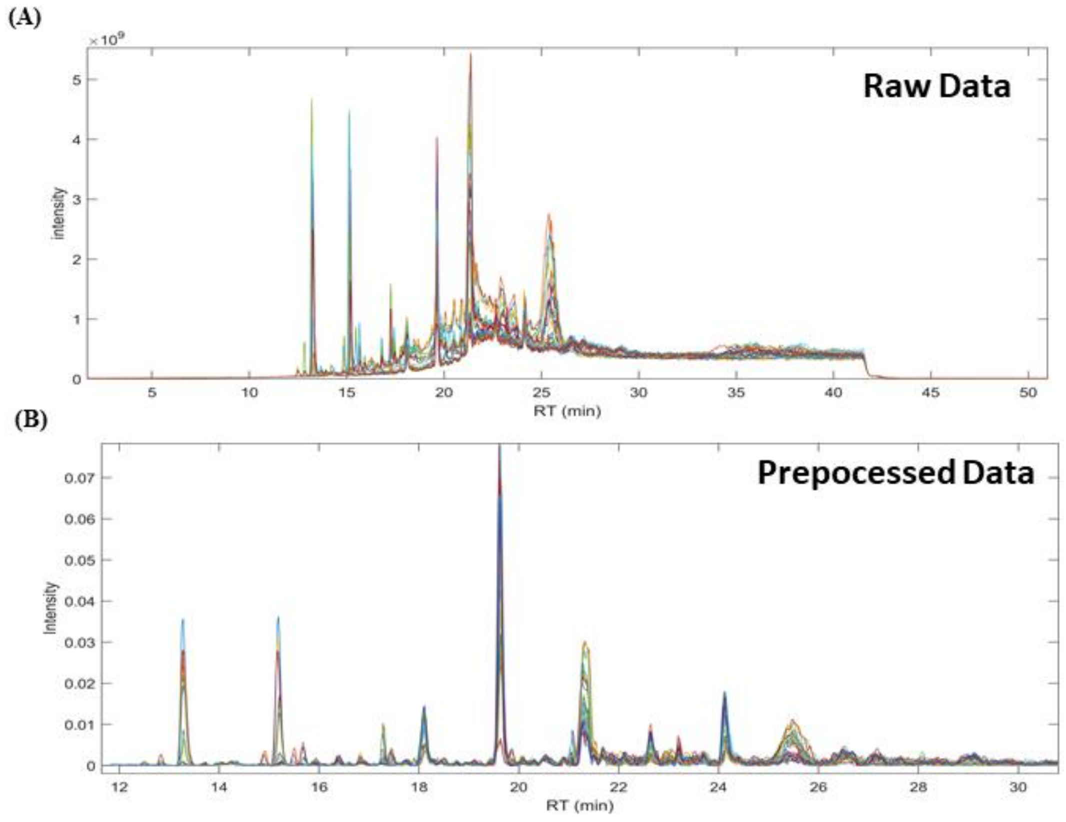

Figure SM4. (A) Total Ion Chromatogram (TIC) of the raw data (before preprocessing). Many different functions and parameters have been tested by trial and error. **(B)** The final matrix was automatically reduced to ASCII files and imported to MATLAB software (Mathworks, Natick, MA). Data preprocessing included the removal of the edges (only data between 11.5 and 31 minutes were used), baseline correction by Automatic Whittaker Filter (asymmetry of 0.001 and lambda 100), variable alignment by peak alignment (maximum shift or slack of 10 and alignment function of linear of 1st order), total area normalization, binning of major peaks (25 bins), G-log transformation (lambda of 10^{-1}) and autoscaling. Autoscaling was done by the mean center values divided by the standard deviation of each variable. It was performed to ensure that all peaks have the same weight (i.e., same importance) in the MDVA analysis.

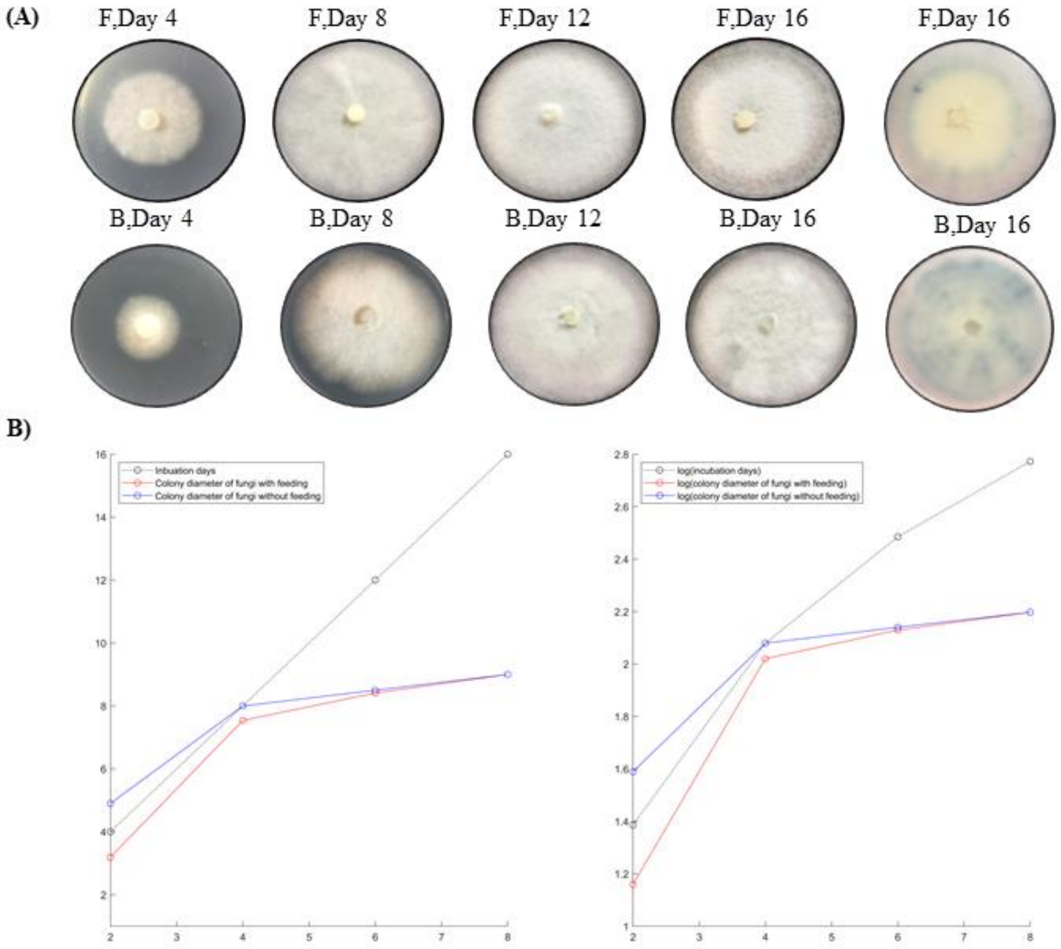

Figure SM5. (A) Petri dishes from the fungal growth with and without the addition of exogenous compounds. Extraction was conducted on days 1, 4, 8, 12, and 16. *Fusarium* samples in the presence of the alkaloids [B] and *Fusarium* control [F] are displayed side by side. Other than the growth rate, visual evaluation of the Petri dishes also revealed that samples in the presence of the alkaloids formed a blue precipitate all over the petri dish from day 4. This same tendency is observed for the control group but at much lower speed and intensity (only at day 16). **(B)** Different treatments of the Y-vector have been tested to accurately represent the microbial metabolic production within a petri dish (plot on the left). The best method was the one that takes into account media availability on different microbial types and the fungi growth rate in the Petri dish. This was achieved by a combination of the colony diameter of the fungi in the petri dish and the log-transformation of these values (plot on the right in red, for the fungi with alkaloidal addition; and in blue for the fungi control without alkaloidal feeding).

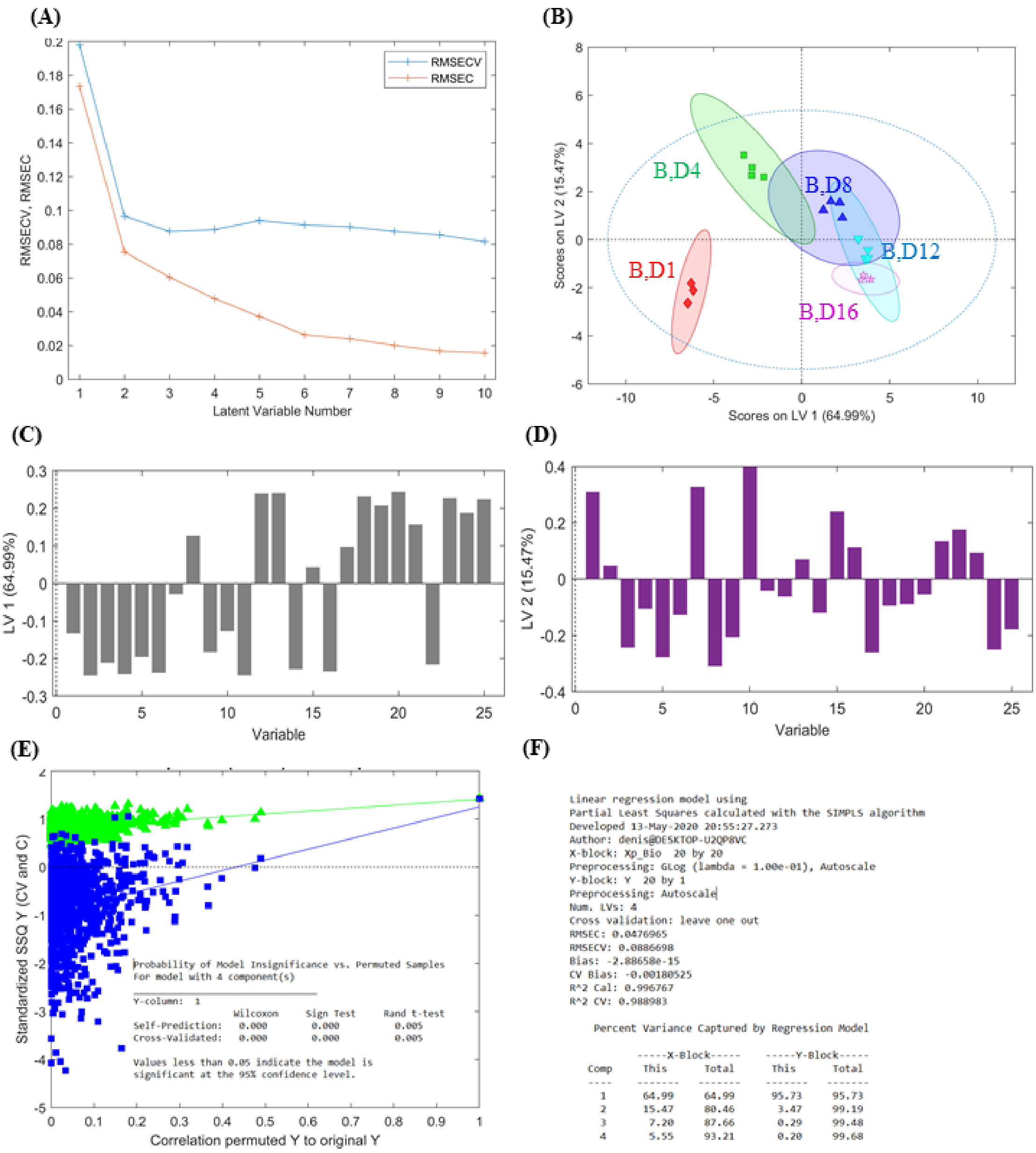

Figure SM6. PLS regression of the *Fusarium* samples with alkaloidal feeding. **(A)** The number of latent variables (LVs) was chosen separately for each data type by the evaluation of the calibration error, which indicates the goodness of the fit between the experimental data and the calibration model. This selection was performed by visual inspection of the Root Mean Squares Error Calibration (RMSEC) and RMSE Cross-Validation (RMSECV) and targeted to select the LV that contains the lowest value for both parameters. The selected LVs for the data with and without alkaloidal addition were, respectively, 4 and 3 LVs. **(B)** PLSr 2D-Score plot of latent variable 1 (variance of 64.99%) and LV2 (variance of 15.47%), with a total variance of 80.46%. Samples were majorly discriminated over LV1, with samples from the late fungi growth (days 8,12, and 16) clustered on the positive LV1 values and early growth sampled (days 1 and 4) grouped on the negative LV1 quadrants. **(C)** PLSr 1D-Loading of LV1 and **(D)** LV2. Loading values show the chemical differences between samples from the early and late growth stages. Bins that are placed on the positive values of the LV1 are correlated to the fungi metabolism, whereas the ones that are shown on the negative LV1 values are correlated to the culture medium as well as alkaloids added to the cultures and biotransformed by the fungi by day 4. **(E)** PLSr was validated by permutation test using 1000 iterations, which was evaluated by three methods (Wilcoxon, sign test, and random t-test), as well as by evaluation of the SSQ_Y plot. P-values below 0.05 were considered statistically significant. This plot shows fractional y-variance captured for self-prediction (calibration) and cross-validation versus the correlation of the permuted y-block to the original y-block. In it, the SSQ_Y, C is expected to increase up to a value of "1" when the model is capturing all y-block responses. The SSQ_Y, CV is expected to be close to SSQ_Y if the model is not over-fit. In general, the cross-validated and self-prediction values should be relatively close to each other but should be significantly less than the results for the non-permuted y-block (blue). **(F)** PLSr was also validated by cross-validation

using the leave one out methodology. The ability to test whether the model could predict the classes (also known as prediction accuracy) was done by the evaluation of the sum of squares captured by the model (R2) and the cross-validation (R2-CV or Q2). Both analyses were validated by both cross-validation and permutation tests using 1000 iterations. It is worth mentioning that cross-validation was applied to choose the optimal model parameters and also to test the predictability of the statistical model, whereas the permutation test was only used to assess the significance of a classification (Broadhurst and Kell 2006). In this sense, it is recommended that both protocols be applied to ultimately provide an objective assessment of the performance and stability of a model, addressing chance-correlations and the risk of overfitting. A brief explanation of validation protocols in metabolomics, and the results of the cross-validation and the permutation tests for both datasets (with and without alkaloidal feeding) can be seen in Figures SM6 and SM7 from the Supplementary Material.

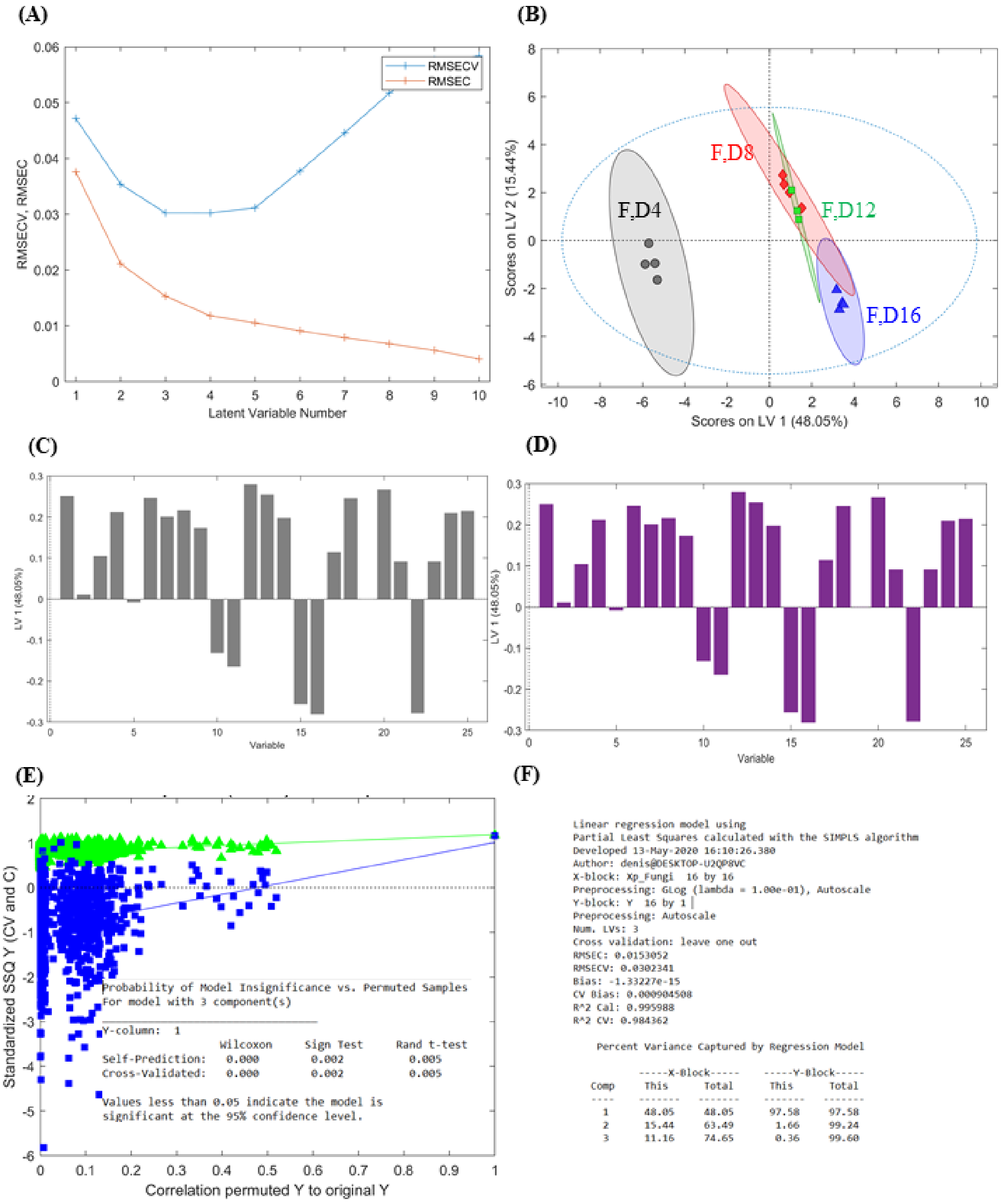

Figure SM7. PLS regression of the *Fusarium* samples without alkaloidal feeding (control). **(A)** The number of latent variables (LVs) was chosen by the evaluation of the calibration error, which indicates the goodness of the fit between the experimental data and the calibration model. This selection was performed by visual inspection of the Root Mean Squares Error Calibration (RMSEC) and RMSE Cross-Validation (RMSECV) and targeted to select the LV that contains the lowest value for both parameters. The selected LVs for the data without feeding were 3 LVs. **(B)** PLSr 2D-Score plot of latent variable 1 (variance of 48.05%) and LV2 (variance of 15.44%), with a total variance of 63.49%. Samples were majorly discriminated over LV1, with samples from the late fungi growth (days 8,12, and 16) clustered on the positive LV1 values and early growth sampled (day 4) grouped on the negative LV1 quadrants. **(C)** PLSr 1D-

Loading of LV1 and **(D)** LV2. Loading values show the chemical differences between samples from the early and late growth stages. Bins that are placed on the positive values of the LV1 are correlated to the fungi metabolism, whereas the ones that are shown on the negative LV1 values are correlated to the culture medium. **(E)** PLSr was validated by permutation test using 1000 iterations, which was evaluated by three methods (Wilcoxon, sign test, and random t-test), as well as by evaluation of the SSQ_Y plot. P-values below 0.05 were considered statistically significant. This plot shows fractional y-variance captured for self-prediction (calibration) and cross-validation versus the correlation of the permuted y-block to the original y-block. In it, the SSQ_Y, C is expected to increase up to a value of "1" when the model is capturing all y-block responses. The SSQ_Y, CV is expected to be close to SSQ_Y if the model is not over-fit. In general, the cross-validated and self-prediction values should be relatively close to each other but should be significantly less than the results for the non-permuted y-block (blue). **(F)** PLSr was also validated by cross-validation using the leave one out methodology. The ability to test whether the model could predict the classes (also known as prediction accuracy) was done by the evaluation of the sum of squares captured by the model (R2) and the cross-validation (R2-CV or Q2).

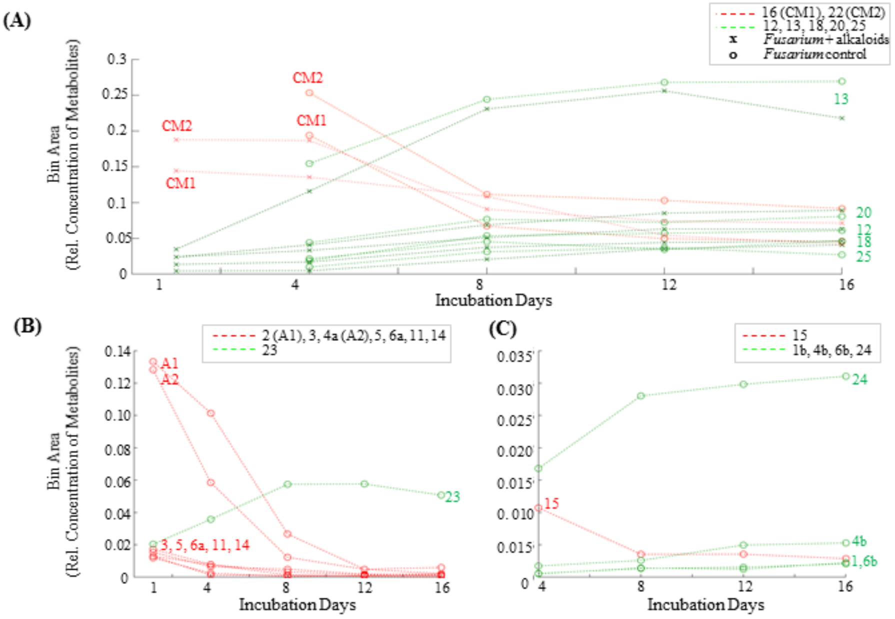

Figure. SM8. Total area plot of bins that were statistically significant on the PLSr analysis for *F. oxysporum* samples with and without alkaloidal feeding. The plots show the mean value of the bin areas on each incubation day and are colored according to their correlation in the PLSr. If the bin area showed a positive correlation, i.e., increasing values with time, the line and dots were colored in green. If the area is decreasing with time (i.e., being consumed), it is colored in red. **(A)** Bins statistically relevant for PLSr of *Fusarium* samples with and without alkaloidal feeding. This represents the bins that were significant on the PLSr of both data types and include peaks from the culture media (bins 16 and 22, shown here as CM1 and CM2, respectively) and others that belong to the standard monoculture metabolism of *F. oxysporum*. **(B)** Bins that were statistically significant only for *Fusarium* samples with alkaloidal feeding. These bins include 2 and 4a, which belong to the alkaloids (-)-cassine (bin 2) and (-)- spectaline (bin 4a), and the metabolites regulated due to the presence of these exogenous compounds. **(C)** Bins are exclusively significant for *Fusarium* control samples. Manual inspection of the LC-MS data showed that these compounds are also present in the fungal samples with alkaloidal feeding. However, appear convoluted with more abundant peaks, hampering their identification and correlation.

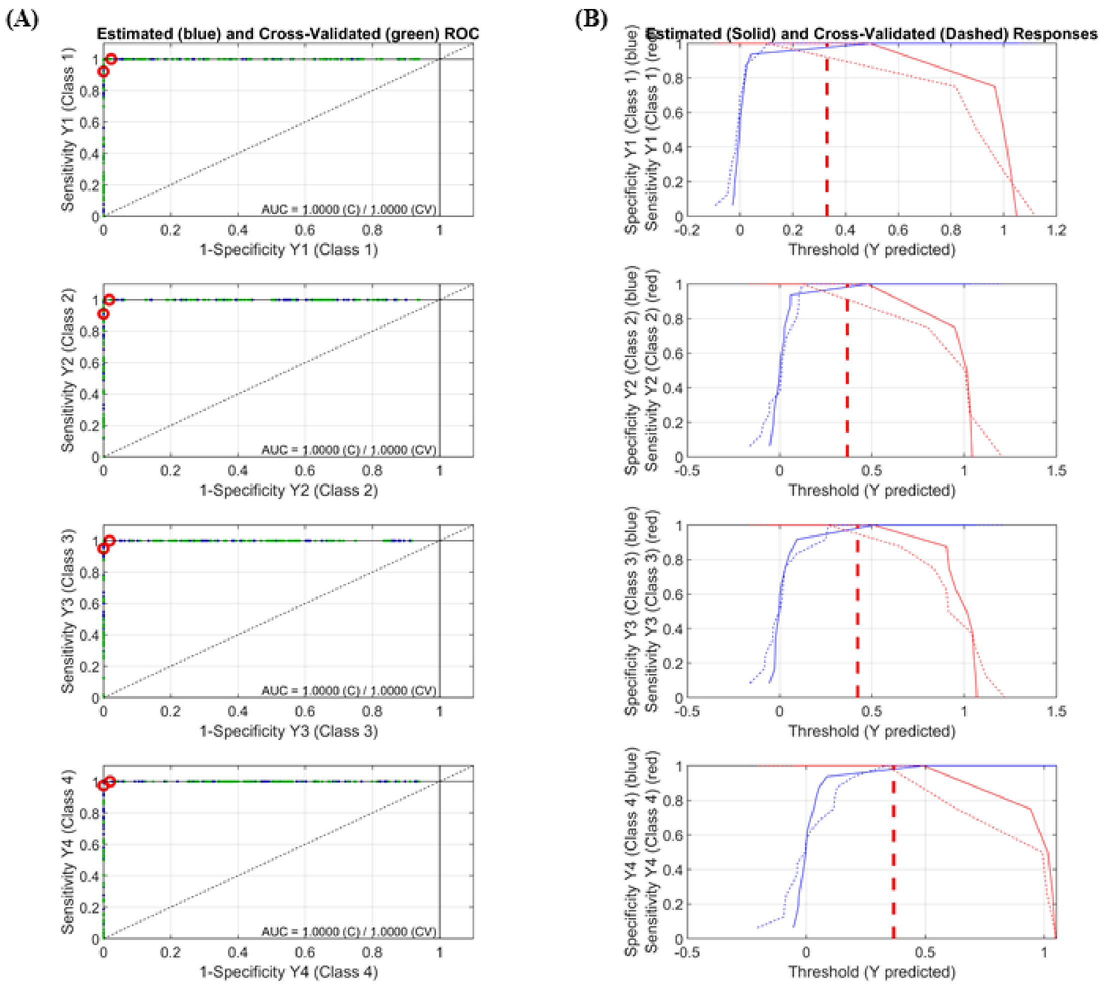

Figure SM9. (A) Receiver operating characteristic (ROC) curves of the PLS-DA model are used to discriminate samples according to incubation days. The red circle identifies selected sensitivity and 1-specificity values for the prediction model. **(B)** The sensitivity/specificity plot of each class curve shows similar information in a different format. The x-axis corresponds to the threshold value used to classify into one group or the other. As you increase the threshold, the specificity increases, i.e. the false positive rate decreases. Likewise, as the sensitivity decreases, the false-negative rate increases.

(A)

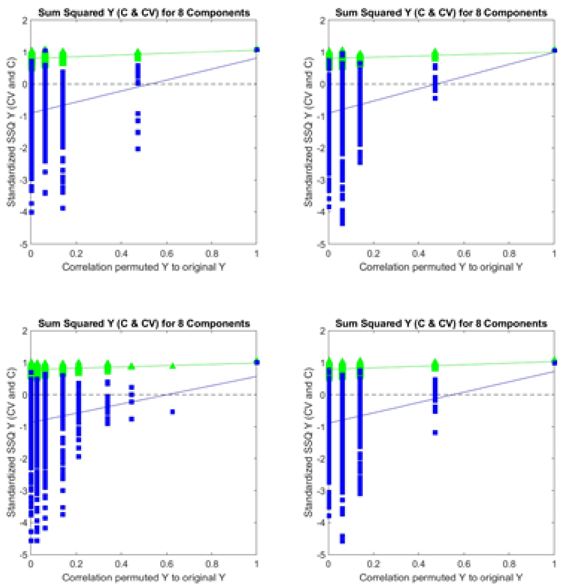


```
Probability of Model Insignificance vs. Permuted Samples
For model with 8 component(s)
_____________________________________________
Y-column:  1
                 Wilcoxon      Sign Test      Rand t-test
Self-Prediction:   0.000         0.001          0.005
Cross-Validated:   0.000         0.001          0.005
_____________________________________________
Y-column:  2
                 Wilcoxon      Sign Test      Rand t-test
Self-Prediction:   0.000         0.005          0.005
Cross-Validated:   0.000         0.004          0.005
_____________________________________________
Y-column:  3
                 Wilcoxon      Sign Test      Rand t-test
Self-Prediction:   0.000         0.004          0.005
Cross-Validated:   0.000         0.002          0.005
_____________________________________________
Y-column:  4
                 Wilcoxon      Sign Test      Rand t-test
Self-Prediction:   0.001         0.008          0.006
Cross-Validated:   0.001         0.012          0.006

Values less than 0.05 indicate the model is
significant at the 95% confidence level.
```

(B)

```
This is a model of type: PLSDA
X-block: Xp_Biof  20 by 20
Preprocessing: GLog (lambda = 1.00e-01), Autoscale
Y-block: Y2  20 by 4
Num. LVs: 8
Cross validation: leave one out

Statistics for each y-block column:
Modeled Class: 1   2   3   4
Sensitivity (Cal):  1.000  1.000  1.000  1.000
Specificity (Cal):  1.000  1.000  1.000  1.000
Sensitivity (CV):  1.000  1.000  1.000  1.000
Specificity (CV):  1.000  1.000  1.000  1.000
Class. Err (Cal):     0     0     0     0
Class. Err (CV):     0     0     0     0
RMSEC: 0.0263453 0.0417963 0.0573019 0.0487539
RMSECV: 0.0671242 0.0964458 0.144226 0.143614
Bias: -1.97065e-15 4.77396e-15 4.44089e-16 -3.33067e-15
CV Bias: -0.0118385 0.00827184 0.00141099 0.00215567
R^2 Cal: 0.995662 0.989082 0.986319 0.985144
R^2 CV: 0.972784 0.945685 0.914478 0.871999

    Percent Variance Captured by Regression Model

             -----X-Block-----     -----Y-Block-----
  Comp      This       Total      This       Total
  ----     -------    -------    -------    -------
    1       63.12      63.12      32.35      32.35
    2       15.65      78.77      29.10      61.46
    3       10.03      88.81      29.90      91.36
    4        3.08      91.89       5.16      96.51
    5        2.37      94.26       0.61      97.13
    6        1.45      95.71       0.92      98.05
    7        1.11      96.82       0.46      98.51
    8        1.03      97.85       0.40      98.91
```

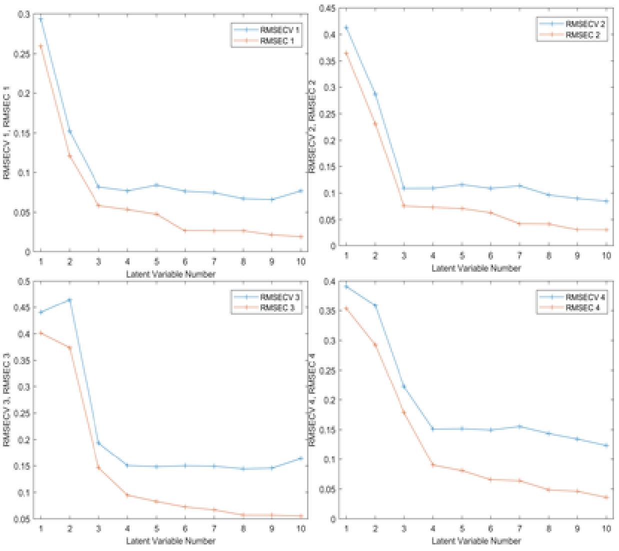

Figure SM10. The classification potential of PLS-DA (model performance) was carefully evaluated by the properties of four diagnostic statistics: (1) the confusion matrix, (2) the area Under the Receiver Operating Characteristic (AUROC), which includes evaluation of specificity, sensitivity, and threshold values, (3) sum of square captured by the cross-validation and (4) the empirical p-values from the permutation tests. Only data statistically validated in all four-performance diagnostic was used for the analysis. Overall, this 4-class model (1, 4, [8/12], 16) achieved the highest sensitivity and specificity, displaying 100% accuracy and 0% error rate. Moreover, a permutation test using 1000 interactions was performed to ascertain whether the separation was due to chance and reveal that, for all classes, the p-values were below 0.05 on three different tests (Wilcoxon Mann–Whitney, Sign, and Rand t-test). **(A)** PLS-DA was validated by permutation test using 1000 iterations, which was evaluated by three methods (Wilcoxon, sign test, and random t-test) for each class, as well as by evaluation of the SSQ_Y plot. P-values below 0.05 were considered statistically significant. This plot shows fractional y-variance captured for self-prediction (calibration) and cross-validation versus the correlation of the permuted y-block to the original y-block. In it, the SSQ_Y, C is expected to increase up to a value of "1" when the model is capturing all y-block responses. The SSQ_Y, CV is expected to be close to SSQ_Y if the model is not over-fit. In general, the cross-validated and self-prediction values should be relatively close to each other but should be significantly less than the results for the non-permuted y-block (blue). **(B)** PLS-DA was also validated by cross-validation using the leave one out methodology. The ability to test whether the model could predict the classes (also known as prediction accuracy) was done by the evaluation of the sum of squares captured by the model (R2) and the cross-validation (R2-CV or Q2).

```
PLSDA Classification Using Rule: Pred Most Probable

MODEL RESULTS
Confusion Matrix:
    Class:      TPR         FPR         TNR         FNR         N      Err         P           F1
   Class 1      1.00000     0.00000     1.00000     0.00000     4      0.00000     1.00000     1.00000
   Class 2      1.00000     0.00000     1.00000     0.00000     4      0.00000     1.00000     1.00000
   Class 3      1.00000     0.00000     1.00000     0.00000     8      0.00000     1.00000     1.00000
   Class 4      1.00000     0.00000     1.00000     0.00000     4      0.00000     1.00000     1.00000

Confusion Table:
                                 Actual Class
                                Class 1  Class 2  Class 3  Class 4
Predicted as Class 1            4        0        0        0
Predicted as Class 2            0        4        0        0
Predicted as Class 3            0        0        8        0
Predicted as Class 4            0        0        0        4
Predicted as Unassigned         0        0        0        0

CV RESULTS
Confusion Matrix (CV):
    Class:      TPR         FPR         TNR         FNR         N      Err         P           F1
   Class 1      1.00000     0.00000     1.00000     0.00000     4      0.00000     1.00000     1.00000
   Class 2      1.00000     0.00000     1.00000     0.00000     4      0.00000     1.00000     1.00000
   Class 3      1.00000     0.00000     1.00000     0.00000     8      0.00000     1.00000     1.00000
   Class 4      1.00000     0.00000     1.00000     0.00000     4      0.00000     1.00000     1.00000

Confusion Table (CV):
                                 Actual Class
                                Class 1  Class 2  Class 3  Class 4
Predicted as Class 1            4        0        0        0
Predicted as Class 2            0        4        0        0
Predicted as Class 3            0        0        8        0
Predicted as Class 4            0        0        0        4
Predicted as Unassigned         0        0        0        0


PLSDA Classification Using Rule: Pred Strict (using strictthreshold = 0.50)

MODEL RESULTS
Confusion Matrix:
    Class:      TPR         FPR         TNR         FNR         N      Err         P           F1
   Class 1      1.00000     0.00000     1.00000     0.00000     4      0.00000     1.00000     1.00000
   Class 2      1.00000     0.00000     1.00000     0.00000     4      0.00000     1.00000     1.00000
   Class 3      1.00000     0.00000     1.00000     0.00000     8      0.00000     1.00000     1.00000
   Class 4      1.00000     0.00000     1.00000     0.00000     4      0.00000     1.00000     1.00000

Confusion Table:
                                 Actual Class
                                Class 1  Class 2  Class 3  Class 4
Predicted as Class 1            4        0        0        0
Predicted as Class 2            0        4        0        0
Predicted as Class 3            0        0        8        0
Predicted as Class 4            0        0        0        4
Predicted as Unassigned         0        0        0        0

CV RESULTS
Confusion Matrix (CV):
    Class:      TPR         FPR         TNR         FNR         N      Err         P           F1
   Class 1      1.00000     0.00000     1.00000     0.00000     4      0.00000     1.00000     1.00000
   Class 2      1.00000     0.00000     1.00000     0.00000     4      0.00000     1.00000     1.00000
   Class 3      1.00000     0.00000     1.00000     0.00000     8      0.00000     1.00000     1.00000
   Class 4      1.00000     0.00000     1.00000     0.00000     4      0.00000     1.00000     1.00000

Confusion Table (CV):
                                 Actual Class
                                Class 1  Class 2  Class 3  Class 4
Predicted as Class 1            4        0        0        0
Predicted as Class 2            0        4        0        0
Predicted as Class 3            0        0        8        0
Predicted as Class 4            0        0        0        4
Predicted as Unassigned         0        0        0        0
```

Figure SM11. Confusion matrix of PLS-DA classification of *Fusarium* with alkaloidal feeding according to the Y-vector (1, 4, 8/12, 16 days of incubation).

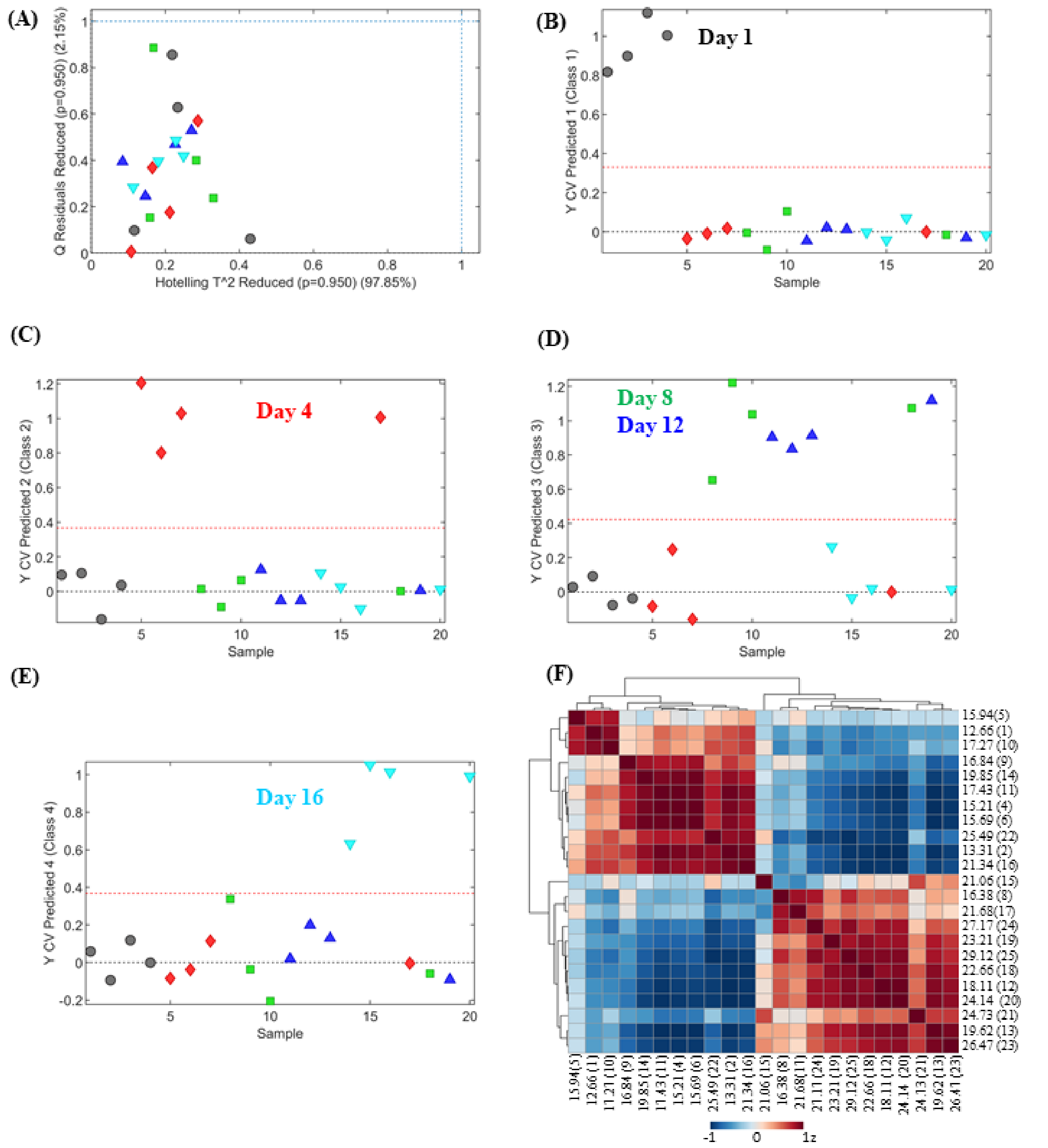

Figure SM12. PLS-DA classification plots according to the *Fusarium* samples with alkaloidal feeding. (**A**) Q residuals vs Hoteling T^2 reduced plot for the detection of outliers. No outliers were detected. **(B)** class 1 (day 1) vs. other classes; (**C**) class 2 (day 4) vs. other classes; (**D**) class 3 (days 8, 12) vs. other classes **(E)** class 4 (days 16). For plots B-E, the red dashed lines indicate the classification boundary. Samples belonging to the targeted class are located on the top, while samples belonging to the other two sample groups were located to the bottom. **(F)** Correlation between variables of the dataset. The statistical relationship between two variables is referred to as their correlation. A correlation could be positive (red), meaning both variables move in the same direction, or negative (blue), meaning that when one variable increases, the other variables decrease.

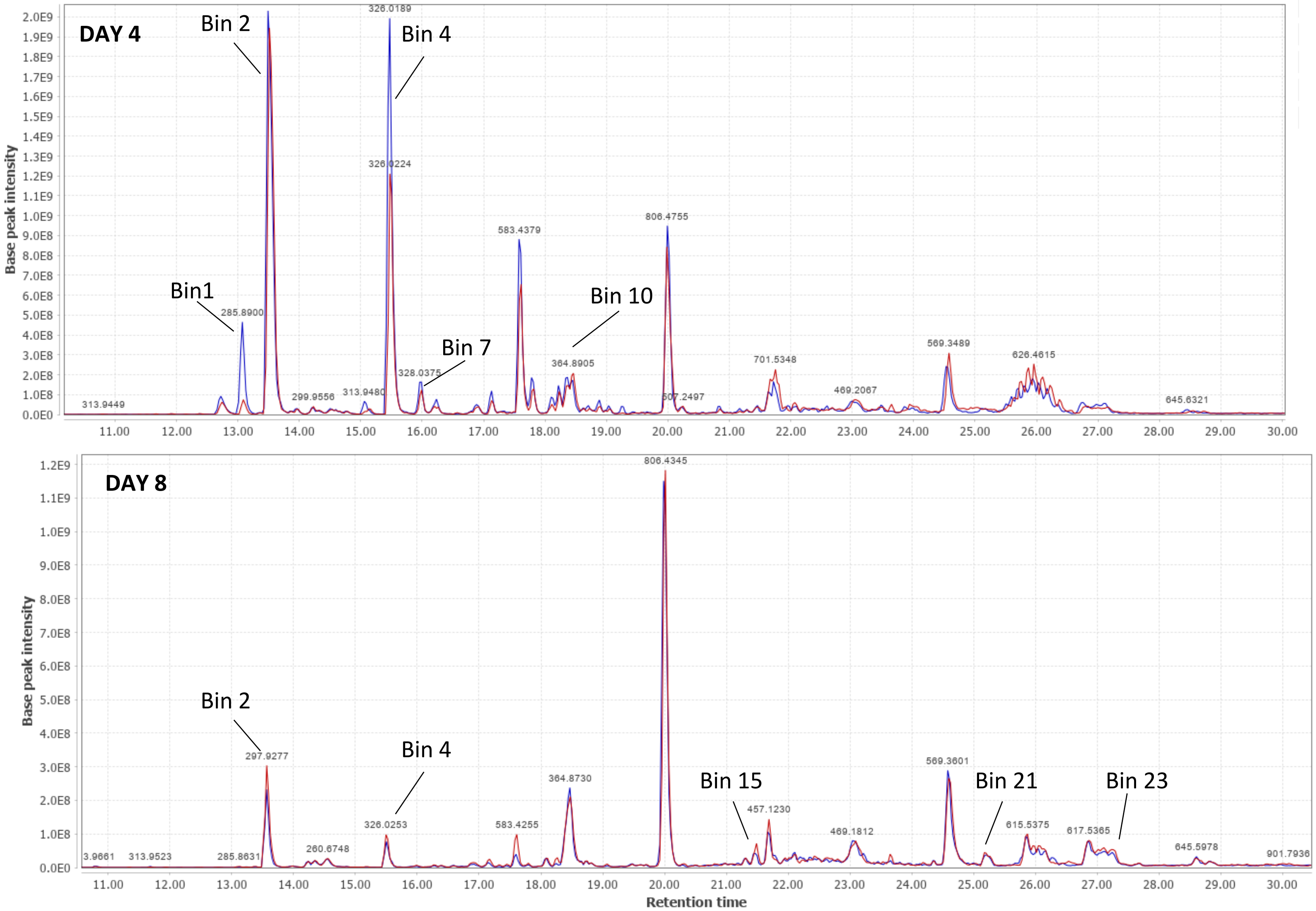

Figure SM13. (A) TIC Chromatogram of (1) day 4 and (2) day 8-12. Bins significant for the chemometric analysis are displayed accordingly.

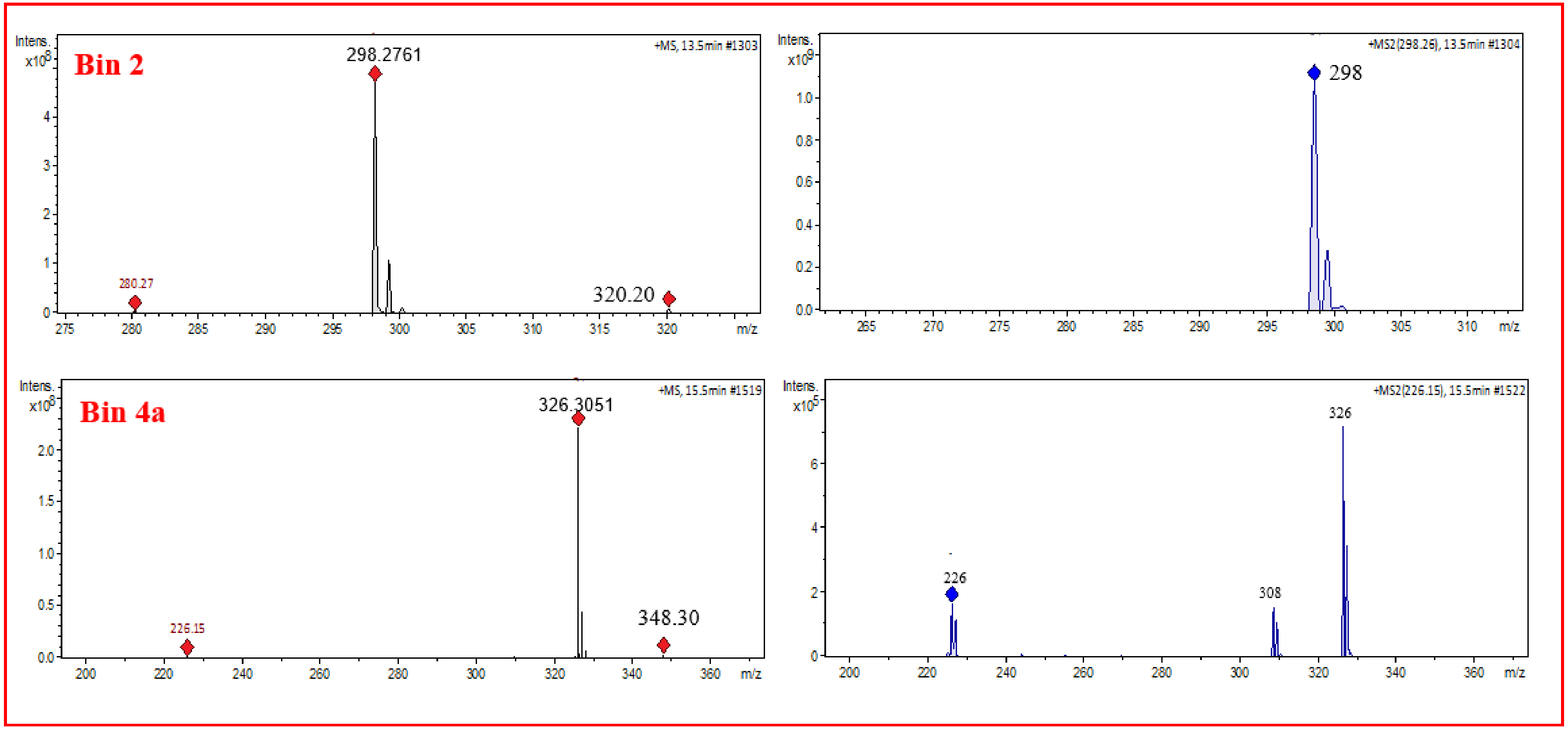

Figure SM13. (B) MS spectra of bins from the piperidine alkaloids (-)-cassine (bin 2, *m/z* 298) and (-)-spectaline (bin 4, *m/z* 326), respectively.

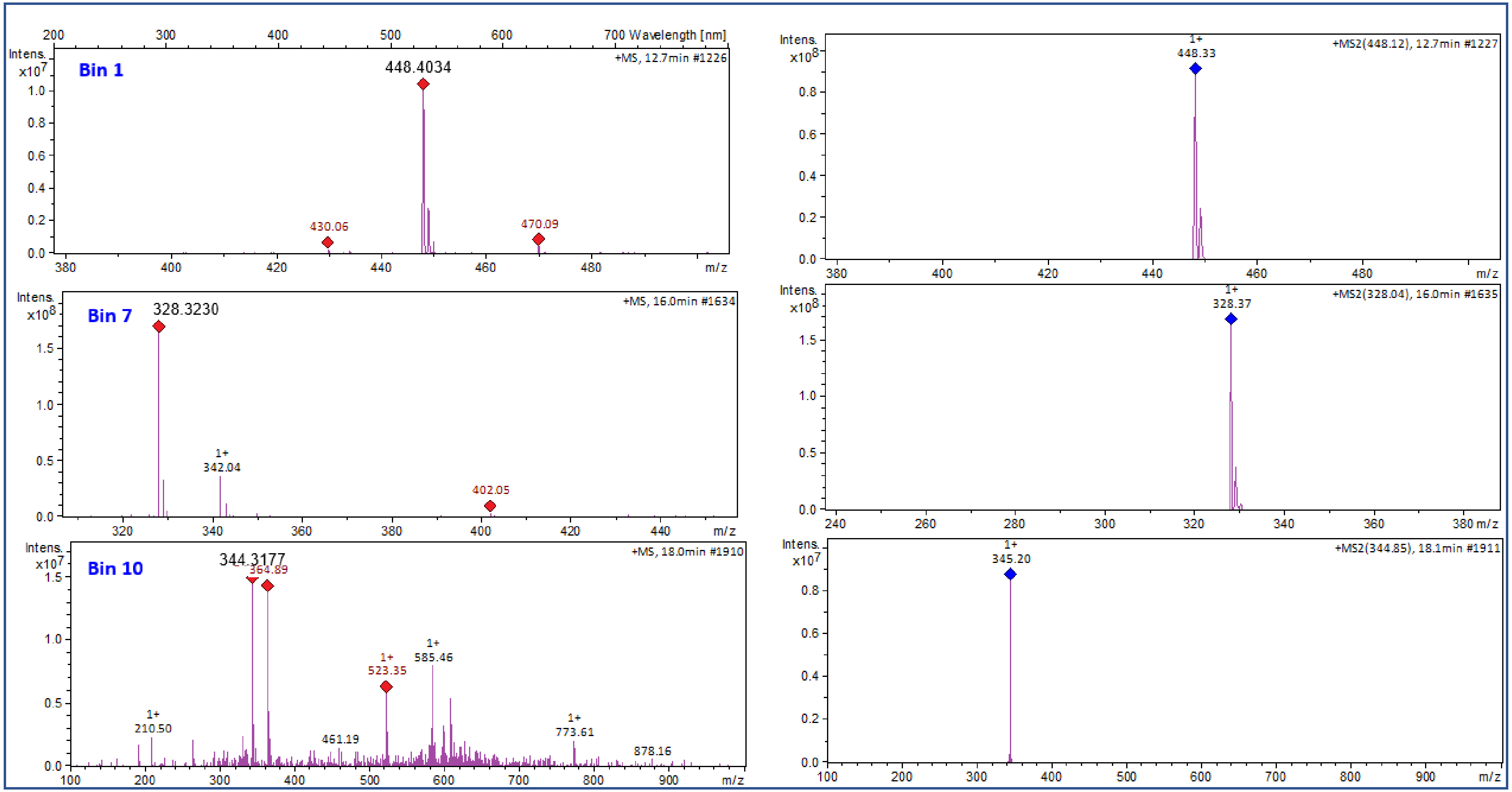

Figure SM13. (C) MS spectra of bins were significantly induced by the piperidine alkaloids on day 4 (bins 1, 7, and 10, respectively).

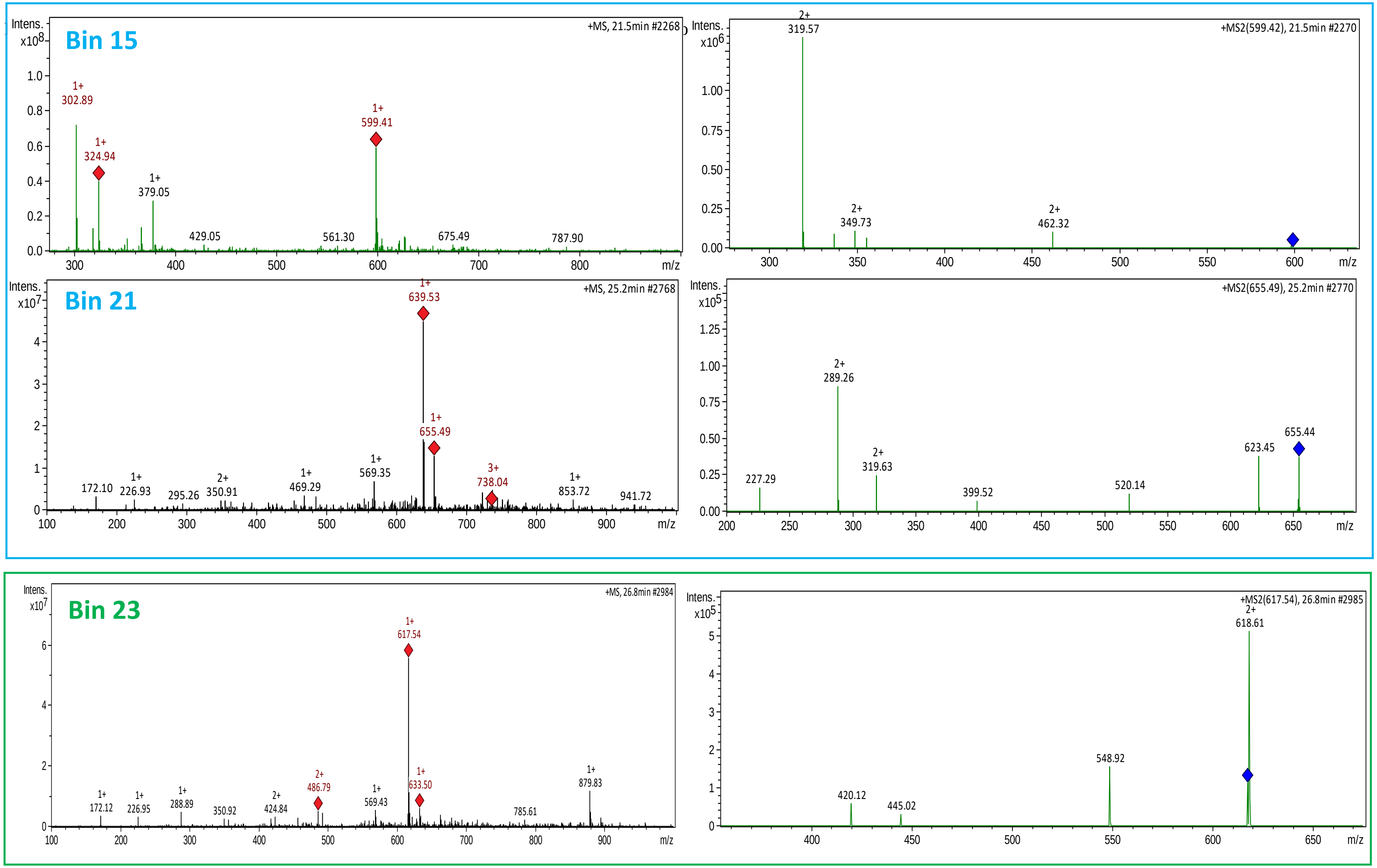
Bin 15
Intens.
x10^8
+MS, 21.5min #2268
1+
302.89
1+
324.94
1+
379.05
429.05
561.30
1+
599.41
675.49
787.90
m/z
Intens.
x10^6
+MS2(599.42), 21.5min #2270
2+
319.57
2+
349.73
2+
462.32
m/z
Bin 21
Intens.
x10^7
+MS, 25.2min #2768
172.10
1+
226.93
295.26
2+
350.91
1+
469.29
1+
569.35
1+
639.53
1+
655.49
3+
738.04
1+
853.72
941.72
m/z
Intens.
x10^5
+MS2(655.49), 25.2min #2770
227.29
2+
289.26
2+
319.63
399.52
520.14
623.45
655.44
m/z
Bin 23
Intens.
x10^7
+MS, 26.8min #2984
1+
172.12
1+
226.95
1+
288.89
350.92
2+
424.84
2+
486.79
1+
569.43
1+
617.54
1+
633.50
785.61
1+
879.83
m/z
Intens.
x10^5
+MS2(617.54), 26.8min #2985
2+
618.61
420.12
445.02
548.92
m/z
